# Effects of 6-months of SSRI use on DNA-methylation and gene expression in blood

**DOI:** 10.1016/j.bbi.2026.106520

**Published:** 2026-02-25

**Authors:** Lauren F Barker, Allan F McRae, Hok Pan Yuen, Anjali K Henders, Leanne M Wallace, Tian Lin, Ella Davyson, Christina Phassouliotis, Jessica Spark, Melissa Kerr, Rebekah Street, Enda M Byrne, G Paul Amminger, Barnaby Nelson, Naomi R Wray, Patrick D McGorry

**Affiliations:** a Institute for Molecular Bioscience, the University of Queensland, Saint Lucia, Queensland, Australia; b Orygen, 35 Poplar Rd, Parkville, VIC 3052, Australia; c Centre for Youth Mental Health, The University of Melbourne, Melbourne, Victoria, Australia; d Division of Psychiatry, Centre for Clinical Brain Sciences, University of Edinburgh, Edinburgh, UK; e Institute of Genetics and Cancer, University of Edinburgh, Edinburgh, UK; f Child Health Research Centre, The University of Queensland, Saint Lucia, Queensland, Australia; g Department of Psychiatry, University of Oxford, Oxford, UK

**Keywords:** DNA-methylation, Gene expression, SSRI, Clinical trial, Inflammation, *GCG*, antidepressants

## Abstract

**Background:**

Selective serotonin reuptake inhibitors (SSRIs) are a recommended first line medication for the treatment of major depressive disorder, due to higher tolerability and lower risk of adverse effects than other antidepressants. The mechanisms by which SSRIs reduce depressive symptoms are not well understood, but are hypothesised to include direct effects on serotonin signalling and synaptic remodelling, and indirect effects on inflammation.

Indirect or off-target effects may be detectable in blood and can be investigated using methylome- and transcriptome-wide approaches.

**Methods:**

The Staged Treatment in Early Psychosis (STEP) clinical trial included a 6-month long randomised, placebo-controlled trial of the SSRI fluoxetine in a cohort of young people at ultra-high risk for psychosis. A methylome-wide association study (MWAS; NTotal (before/after/both) = 104 (52/52/44), NSSRI (before/after/both) = 45 (21/24/18)) and differential expression analysis were performed on longitudinal blood samples collected at the start and end of the 6 months to identify changes in DNA-methylation and gene expression associated with medium-term SSRI exposure.

**Results:**

Four methylation CpGs (*cg26253898*, *cg09719563*, *cg22216017*, *cg26017656*) were significantly associated with SSRI exposure (FDR < 0.1, 2 CpGs at FDR < 0.05) and annotated to genes involved in glucose metabolism, synaptic remodelling and inflammation (*GCG*, *COL23A1*, *PEG10*, *SGCE*, *MFGE8*). Gene-set enrichment analyses of genes annotated to the top 100 CpGs identified significant tissue-specific enrichments in artery, adipose and spleen tissues, and in the ‘postsynaptic density’ GO term. No genes were differentially expressed, including genes annotated to the significant methylation CpGs.

**Conclusions:**

Medium-term SSRI use during the STEP trial was associated with changes in DNA-methylation that may partially explain the potential antidepressant mechanisms and adverse effects of SSRIs, however replication in other cohorts is necessary to establish if these changes are generalisable to SSRI use more broadly.

## 1. Introduction

Major depressive disorder is estimated to affect between 11–15% of Australians during their lifetime (Malhi et al., 2021; Bromet et al., 2011). Current treatments for depression include psychological therapies (e.g. cognitive behavioural therapy) and medication, however, it is estimated that only 67% of patients will find a tolerated treatment and experience remission of depressive symptoms, even after trialling multiple different treatments (Rush et al., 2006).

Although all antidepressant medications are effective at reducing symptoms of depression in placebo-controlled trials, selective serotonin reuptake inhibitors are recommended as first line medications for depression due to their higher tolerability and lower risk of severe adverse effects (Malhi et al., 2021; Lochmann and Richardson, 2019; Malhi et al., 2022; Walker, 2013). SSRIs are usually well-tolerated (Lochmann and Richardson, 2019; Cipriani et al., 2018) and individuals prescribed an SSRI typically persist with treatment longer than those prescribed other classes of antidepressants, including MAOIs and TCAs (Malhi et al., 2022). However, despite a lower risk of adverse effects than their predecessors, SSRIs still have a significant risk of adverse effects relative to placebo, including nausea, dizziness, insomnia, tremor and sexual dysfunction (Walker, 2013).

The accepted primary mechanism by which SSRIs affect depressive symptoms is by modifying serotonin signalling (Walker, 2013; Stahl, 2000). SSRIs bind with high affinity to the serotonin transporter (SERT, alternatively 5-HTT), thus reducing the re-uptake of serotonin into the pre-synaptic cell and increasing its concentration in the synaptic gap (Walker, 2013). However, although these compounds were chosen based on their selectivity for SERT, all of them still bind at varying levels to the norepinephrine transporter (Owens et al., 1997) and many will also impact other neurotransmitter receptors, including cholinergic and adrenergic receptors (Walker, 2013; Owens et al., 1997). SSRIs also inhibit the activity of cytochrome *P*-450 enzymes to varying degrees, increasing the risk of adverse interactions with other drugs (Lochmann and Richardson, 2019; Hiemke and Hartter, 2000).

The development of SSRIs was based on the monoamine hypothesis of depression, which posited that depression and depressive symptoms were due to a deficiency in one or more monoamines (Hindmarch, 2001). This hypothesis has since been heavily questioned and revised (Walker, 2013; Stahl, 2000), due in part to high non-response rates to SSRIs and, in those who respond, the lag between the fast action of SSRIs in increasing extracellular serotonin compared to the much slower action in improving depressive symptoms (Hindmarch, 2001; Lacasse and Leo, 2005). This has led some to hypothesise that the mechanism by which SSRIs improve depression is not due to its direct effect on the serotonin transporter but, rather, due to indirect effects, such as on inflammation (Lochmann and Richardson, 2019; Hindmarch, 2001; Martensson and Nassberger, 1993). SSRIs have exhibited inhibitory effects on inflammatory cytokines and cell-types (Martensson and Nassberger, 1993; Diamond et al., 2006; Taler et al., 2007), including in models of neuroinflammation (Tynan et al., 2012; Czeh and Di Benedetto, 2013). Additionally, there are demonstrated effects of SSRIs reducing inflammation in non-psychiatric inflammatory disorders (Sacre et al., 2010). However, although the anti-inflammatory effect of SSRIs is well established, the relationship between decreased inflammation and attenuation of depressive symptoms is not well understood, as the reduction in inflammation may occur regardless of changes in depressive symptoms (Kohler et al., 2018).

There is, therefore, a need to better understand the mechanisms behind SSRIs, in order to both better explain how they mitigate depressive symptoms and to understand the causes of unintended and adverse effects. DNA-methylation and gene expression may provide insights into this, as they are both highly dynamic and are susceptible to, and reflective of, changes in environment, including medications. In addition, as the mechanisms behind both the efficacy and adverse effects of SSRIs are not fully known, using methylome- and transcriptome-wide approaches for these analyses may allow for the identification of novel genes and pathways.

Previous analyses of DNA-methylation associations with SSRI use have largely been restricted to candidate genes/regions or have focused on differences in methylation between antidepressant responders and non-responders (Webb et al., 2020). These have confirmed differential methylation in key genes known, or suspected, to be involved in the action of SSRIs, including *SLC6A4*, the gene encoding SERT (Moon et al., 2023), and *BDNF*, which is heavily involved in synaptic plasticity (Pathak et al., 2022; Martinowich and Lu, 2008). To date, there have only been two large MWAS on antidepressant exposure (the second an extension of the first) (Barbu et al., 2022; Davyson et al., 2025). Both MWAS were performed in blood-derived DNA-methylation samples from Generation Scotland and used self-reported and prescription-derived antidepressant use. The first identified 10 CpGs associated with self-reported antidepressant use and found significant enrichment of signal in gene-sets associated with the innate immune response (Barbu et al., 2022). The second identified 7 significant CpGs for self-reported antidepressant use and 4 associated with prescription-derived antidepressant use (Davyson et al., 2025). This included two probes, annotating to *DGUOK-AS1* and *KANK1*, for which a significant correlation existed between the length of time on the antidepressant and the level of DNA-methylation at that probe.

Additionally, there have been several methylome-wide association studies of pre-natal SSRI exposure via maternal SSRI usage, which have identified a small number of associations with DNA methylation in genes involved in the glucocorticoid stress pathway and key brain development genes (Kallak et al., 2021), with suggestive associations with DNA-methyltransferase 3 alpha (Non et al., 2014).

Differential expression studies have also largely focused on candidate genes and have not compared effects of SSRIs using non-SSRI control groups (Lin and Tsai, 2016; Menke, 2013). However, there have been several differential expression studies for SSRI exposure in animal models, most of which focus on brain tissues (Rayan et al., 2022; Kumar et al., 2019). These studies have identified a high degree of tissue specificity for genes affected by SSRI exposure and, in particular, have highlighted tissue-specific expression in brain regions that are centres for monoaminergic synthesis and release (Rayan et al., 2022).

There is a significant gap in the literature for a randomised, placebo-controlled trial to assess the effects of SSRIs on DNA-methylation and gene expression in humans. The Staged Treatment in Early Psychosis (STEP) trial was a sequential, multi-stage clinical trial aimed at preventing psychosis and other adverse outcomes in a transdiagnostic cohort of young people at high risk for developing psychosis (McGorry et al., 2023; Hartmann et al., 2022; Nelson et al., 2018). The trial was comprised of three stages of interventions of increasing intensity, with the final stage consisting of a 6-month long placebo-controlled, randomised clinical trial of the SSRI fluoxetine. In this analysis, we utilise the design of the final stage of the trial to examine the effect of six months of SSRI use, by analysing longitudinal changes in DNA-methylation and gene expression in blood.

## 2. Methods

### 2.1. Clinical trial

**Fig. 1.**
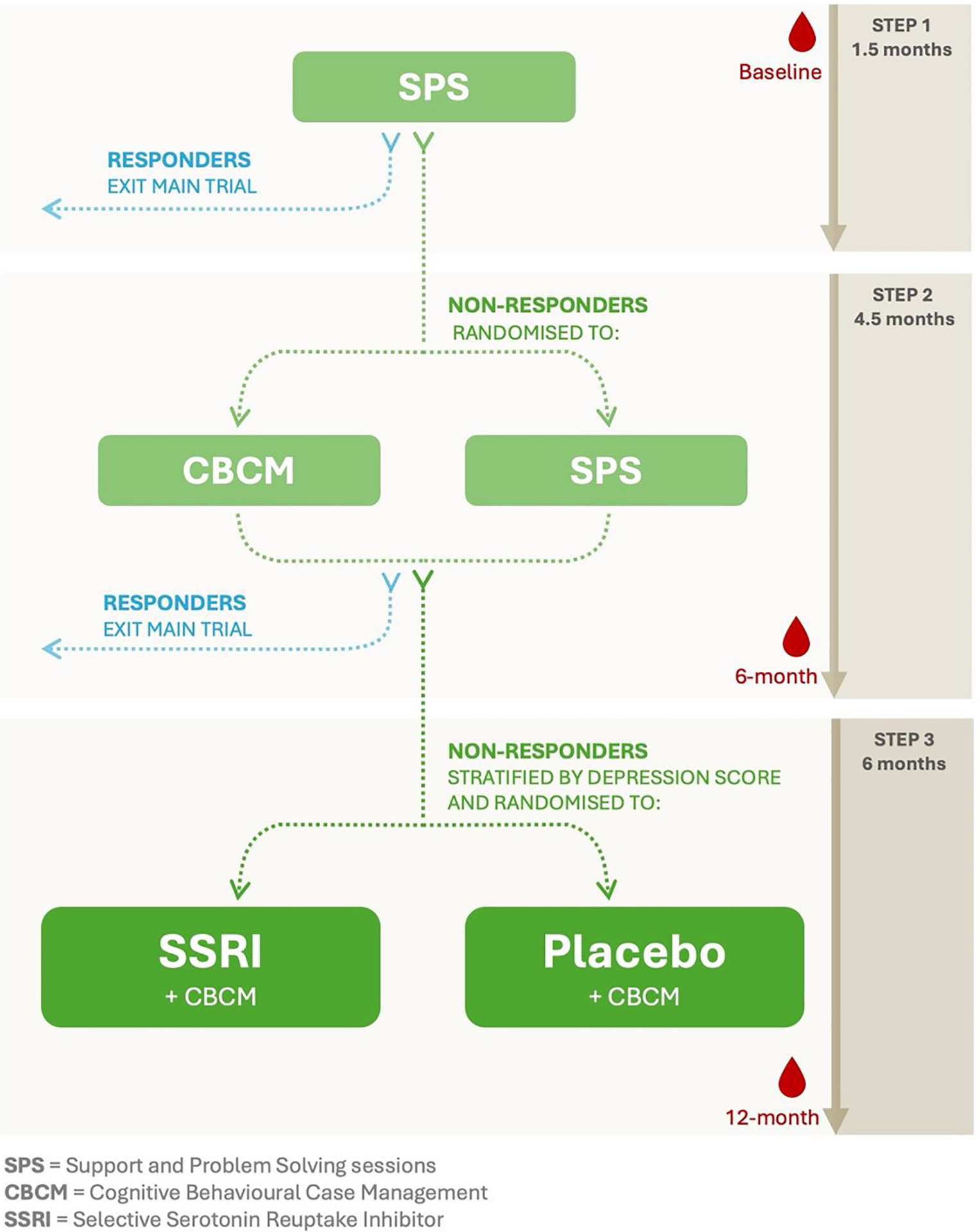
Simplified study design of the main Staged Treatment in Early Psychosis (STEP) clinical trial. Red droplets represent timing of blood sample collection, which was used to generate genotype, DNA-methylation and gene expression data. The analyses in this paper focus on the third step of the trial (SSRI vs Placebo). For analyses focused on steps 1 & 2, please refer to McGorry et al. (2023) for main trial results and to Barker et al. (2025) for analysis of DNA-methylation and gene expression.

The Staged Treatment in Early Psychosis (STEP) clinical trial (ClinicalTrials.gov: NCT02751632) was designed to evaluate the effectiveness of a sequential intervention strategy for preventing psychosis in young people at ultra-high risk (UHR) of developing psychosis (McGorry et al., 2023; Hartmann et al., 2022). Enrolment occurred between April 2016 and January 2019 and was approved by the Melbourne Health Human Research Ethics Committee. Briefly, the study used a clinical staging model (Fig. 1), the first two stages of which comprised two different forms of psychosocial therapy. Participants received open label support and problem solving (SPS) for the first 6 weeks. Those who did not meet remission criteria after stage 1 were randomised to either ongoing SPS or cognitive behavioural case management (CBCM) for the following 20 weeks. Participants were assessed for remission of UHR status at 4 and 6 weeks for stage 1 and at 12 and 24 weeks for stage 2. Participants were required to meet remission criteria at both assessments for a given stage to be considered as remitters. Young people (aged 12–25 years) who met UHR psychosis criteria were recruited from the PACE clinic and four *headspace* youth mental health centres in Melbourne, Australia. Participants were excluded if they had experienced a previous, extended psychotic episode (> 1 week), were diagnosed with neurological, developmental, metabolic or other physical condition (s) known to cause psychosis, or were unable to speak adequate English or provide informed consent. As this trial was transdiagnostic by design, participants were not excluded if they had a pre-existing psychiatric diagnosis. Participants who did not respond to the first two stages of the trial were eligible to enter the third stage, during which they would receive CBCM alongside either fluoxetine or a placebo.

### 2.2. Assessments

UHR status was established using the Comprehensive Assessment of At-Risk Mental States (CAARMS) (Yung et al., 2005), Social and Occupational Functioning Assessment Scale (SOFAS), SCID-II Schizotypal PD, and Family History Index (Nelson et al., 2018; Maxwell, 1992). Participants also completed a self-reported childhood trauma questionnaire (CTQ), as well as the MADRS (Montgomery–Åsberg Depression Rating Scale) (Montgomery and Asberg, 1979), Global Functioning: Social and Role scales (Cornblatt et al., 2007), Brief Psychiatric Rating Scale (BPRS) (Overall and Gorham, 1962), Scale for the Assessment of Negative Symptoms (SANS) (Andreasen, 1989), and Davos Assessment of Cognitive Biases Scale (DACOBS) (van der Gaag et al., 2013). Additionally, participants underwent diagnostic testing at baseline to establish if they met criteria for any psychiatric diagnoses. Participants entering the third stage of the trial were screened for medical safety reasons, including receiving blood tests from an accredited pathology service.

STEP trial participants were given the option to consent for participation in genomic analyses. Blood samples from consenting participants were used to generate genomics data, including genotypes, DNA methylation profiles and gene expression profiles. The ‘bio-cohort’ who provided one or more of these samples did not differ from the main trial cohort for most demographic or clinical measures at trial baseline (Supplementary Tables 1–2). Generation of genomic data was conducted under Human Research Ethics governance of the University of Queensland.

### 2.3. Inclusion criteria

Participants were included in the following analyses if they completed the third stage of the STEP trial (i.e. did not withdraw or cease the intervention early), had a valid 6- or 12-month DNA-methylation or gene expression sample, and had a genotype sample for inference of genetic ancestry. Six-month samples were removed if the participant had been randomised to receive the SSRI and reported beginning their trial medication prior to providing the 6-month sample.

### 2.4. Statistical analysis

#### 2.4.1. Univariate testing of demographic and clinical variables on treatment group

Differences in demographic variables at the 6-month timepoint were tested using a Wilcoxon–Mann–Whitney test (continuous variables) or Pearson’s chi-squared test (non-continuous variables).

#### 2.4.2. Association of clinical measures with SSRI exposure

Linear regression models were used to assess the effect of SSRIs on the following clinical measures: MADRS, total CAARMS, BPRS, SOFAS, SANS, Global functioning (role and social) and DACOBS. All participants who provided at least one DNA-methylation/gene expression sample were included in the analysis. SSRI exposure was modelled as the interaction between timepoint and treatment group. Sex, age and two genetic PCs (for inference of ancestry) were included as covariates and participant was included as a random effect. A Bonferroni-corrected p-value threshold of 6.25 × 10^−3^ was used to declare significance.

### 2.5. Preparation of DNA methylation data

Methylation profiling was performed on whole blood samples using the Infinium MethylationEPIC array (v1) and QC and normalisation were performed with the R package Meffil, using recommended parameters (Min et al., 2018). Full details of data generation and QC can be found in the Supplementary Methods. Cell-type proportions were estimated using the Houseman method (Houseman et al., 2012) as implemented in the *meffil* package, with cell-specific methylation profiles from Reinius et al. (Reinius et al., 2012) used as the reference dataset.

Non-autosomal probes were removed (Shen et al., 2025; Mulder et al., 2021). Singular value decomposition analysis, as implemented in the *Champ* package (Tian et al., 2017), was then used to identify batch effects that were not fully normalised out during QC and *ComBAT* (Leek et al., 2012) was subsequently used to remove the effect of array slide.

#### 2.5.1. MWAS

Methylome-wide association studies (MWAS) were performed to identify CpG sites associated with taking an SSRI (fluoxetine). MWAS analysis was performed using limma (Ritchie et al., 2015) on ComBAT-corrected methylation betas. SSRI exposure was modelled as the interaction between timepoint and treatment group and sex, age, two genetic PCs (for inference of ancestry), methylation-derived cell-type proportions, and methylation-predicted smoking score were included as covariates. To account for repeated measures across timepoints, participant ID was included as a random effect using limma’s *duplicateCorrelation* function.

MWAS was performed using all eligible 6- and 12-month samples, including from participants who provided a methylation sample at only one timepoint (for MWAS performed using only participants with both timepoints, see the Supplementary Text and Supplementary Figs. 2 and 3). Probes with a Benjamini-Hochberg-adjusted p-value less than 0.05 were considered significant and adjusted p-values less than 0.1 were considered suggestive of significance.

Sensitivity analyses on BMI and age can be found in the Supplementary Results.

### 2.6. Follow-up of MWAS results

Genes, UCSC CpG Island locations and probe positions were annotated using the Infinium MethylationEpic manifest. Phenotypes previously associated with significant probes were identified using the MRC-IEU EWAS catalog (Battram et al., 2022). Significant probes were queried in GoDMC’s mQTLdb (Min et al., 2021) and in the Framingham heart study eQTM dataset (Keshawarz et al., 2023). For significant probes, the BeCON shiny app was used to identify correlations between methylation in the blood and brain (Edgar et al., 2017).

The top 100 probes for SSRI exposure were taken forward for functional follow-up. Genes were annotated to probes using the infinium MethylationEpic manifest. Tissue enrichment analyses were performed with the GENE2FUNC tool in FUMA (Watanabe et al., 2017) against GTEX V8 and BrainSpan tissue samples (Kang et al., 2011; Consortium and G, 2020). Enrichment was performed for synapse-related GO-terms using the SynGO web tool (Koopmans et al., 2019). To examine the overlap with major depressive disorder (MDD), the top probes for SSRI exposure were compared against a recent large MWAS of MDD (Shen et al., 2025) (Supplementary Fig. 8).

### 2.7. Differential expression

Differential expression analysis was performed to identify genes that were differentially expressed in response to taking an SSRI (fluoxetine). As in the MWAS, SSRI exposure was modelled as the interaction between timepoint and treatment group and sex, age, sequencing pool and row, two genetic PCs and methylation-derived cell-type proportions were included as covariates. Pre-filtering of genes was performed using the *filterByExpr* function in the *edgeR* (Chen et al., 2016) package, which removes genes for which significance of differential expression cannot be accurately assessed due to consistently low expression in a high number of samples (as determined by the design matrix). Differential expression analysis was then performed using a standard *limma-voom* workflow (Law et al., 2014). Genes with a Benjamini-Hochberg-adjusted p-value less than 0.05 were considered differentially expressed. Full details of RNA preparation, sequencing and QC can be found in the Supplementary Methods.

### 2.8. Methylation prediction score of antidepressant exposure

Methylation prediction scores (MPS) were generated using probe weights for the antidepressant exposure MPS previously created and published by Davyson et al. (2025). Of the 212 probes in the Davyson MPS, 209 were present in the cleaned STEP DNA-methylation dataset (Supplementary Fig. 17). Per-sample MPS were calculated as the sum of the beta multiplied by the weight. A one-sided paired *t*-test was used to assess whether the MPS of participants who received the SSRI was significantly greater in post-treatment samples compared to pre-treatment samples. Accordingly, only participants who provided both the 6- and 12-month timepoints were included for this analysis.

A within-participant model was used to test for associations between the antidepressant MPS and clinical measures, using the subset of participants who provided both 6- and 12-month timepoints. Full details can be found in the Supplementary Methods.

## 3. Results

### 3.1. Sample characteristics

A total of 60 participants provided eligible samples from either timepoint, 44 of which provided both a 6-month and a 12-month sample (Total n = 104; Table 1). Treatment groups were equivalent for demographic characteristics (Table 1). Participants randomised to the SSRI condition had nominally poorer scores for psychosis-related measures (higher total CAARMS, BPRS and DACOBS; Table 1) at 6-months, i.e. prior to starting the SSRI, however the differences were not significant after correction for multiple testing.

In accordance with the findings of the main trial cohort (McGorry et al., 2023), treatment group was not associated with any change in symptoms over the 6-months of treatment at either a nominal or multiple-testing-corrected significance threshold (Supplementary Table 4), despite the initial differences in clinical scores prior to starting treatment. Similarly, no association between white blood cell proportions or counts and treatment was identified (Supplementary Fig. 20.

#### 3.1.1. MWAS

**Fig. 2.**
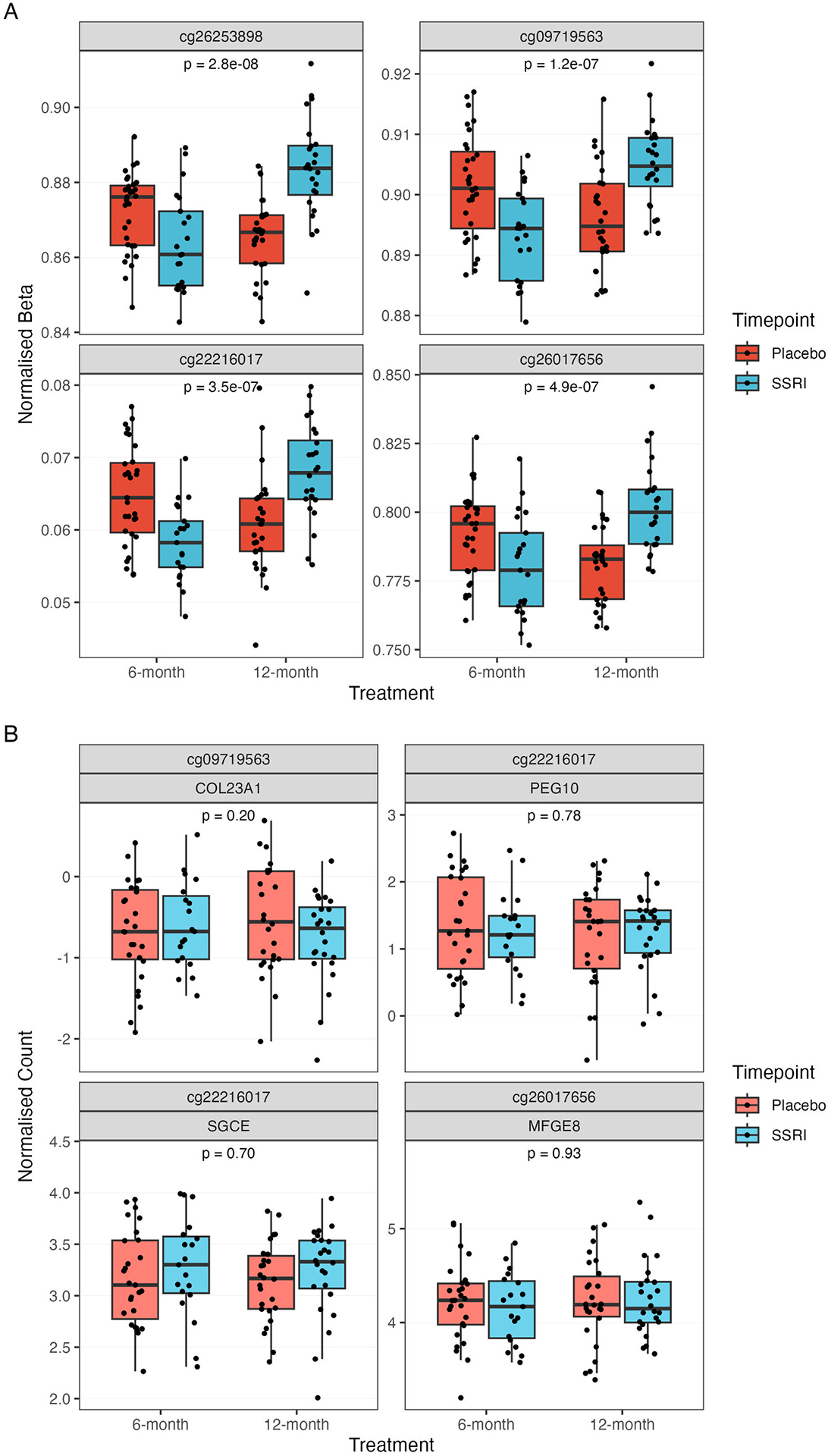
A) DNA-methylation beta values for CpGs significantly associated with SSRI exposure. B) Limma-voom-normalised expression values for genes annotated to significant probes for SSRI exposure. No genes were significantly differentially expressed. The p-value shown on the plot refers to the unadjusted P-value of association with the Treatment:Timepoint interaction term in either the MWAS (A) or differential expression analysis (B). GCG, the gene annotated to the most significant probe (cg26253898), had no expression detected in 93/96 of the samples included for analysis, and was filtered out prior to performing differential expression.

MWAS were performed to identify CpGs that were differentially methylated after taking the SSRI compared to placebo. We identified two CpGs with an FDR-adjusted p-value less than 0.05, and a further two with FDR less than 0.1 (Table 2, Fig. 2.A). For all four of these probes, the significant interaction between timepoint and treatment is characterised by (non-significant) lower DNA-methylation in the SSRI group than the placebo group pre-treatment, but higher DNA-methylation for SSRI than placebo post-treatment (Fig. 2.A, Supplementary Table 5).

Previous MWAS associations with these probes are shown in Table 2. The significant probes did not have any known mQTLs, eQTMs or previous associations with SSRI usage or depression-related phenotypes (Table 2, Supplementary Fig. 8). As the brain is expected to be the tissue of most relevance for SSRI treatment, BeCON (Edgar et al., 2017) was used to identify correlations between brain and blood methylation for the two significant probes present in its database. *cg26253898* had a moderate negative correlation between blood and Brodmann area 10 (R = −0.54; Supplementary Table 7). No other meaningful correlations were reported (Supplementary Figs. 9 and 10).

### 3.2. Follow-up analyses

Of the top 100 CpGs associated with SSRI exposure, 66 had at least one annotated gene with a recognised HGNC symbol (78 genes total; Supplementary Table 6). Of these, 77 genes were recognised by FUMA.

Tissue-specific enrichment analysis by FUMA identified significant enrichments of this geneset in adipose, spleen and coronary artery tissues (Supplementary Figs. 11 and 13, Supplementary Table 8), while gene-set enrichment analysis identified a significant enrichment in the MSigDB Hallmark Apical Junction gene-set (P_FDR_ = 0.019). Analysis of synapse-specific GO-terms identified the cellular component terms ‘postsynaptic density, intracellular component’ (GO:0099092, P_FDR_ = 0.012).

A sensitivity analysis performed to assess whether changes in BMI may be responsible for the significant enrichment in adipose tissue, found that the inclusion of BMI as a covariate did not meaningfully affect the association between DNA-methylation and SSRI use for the top 100 CpGs (Supplementary Fig. 7).

#### 3.2.1. Differential expression

Differential expression analysis was performed to identify genes that are differentially expressed (differentially expressed gene; DEG) in response to taking an SSRI. There were fewer eligible gene expression samples compared to DNA-methylation samples, driven primarily by participants who had provided both timepoints for DNA-methylation but only one timepoint for gene expression (total n = 96; Supplementary Fig. 14). No differentially-expressed genes were identified after correction for multiple testing. Of the genes annotated to the 4 significant MWAS CpGs, one gene (*GCG*) had no expression detected in 97% of samples and was not included for DE analysis. The other four genes were not differentially expressed, even when using only a nominal significance threshold of p < 0.05 (Fig. 1).

#### 3.2.2. Methylation prediction score of SSRI exposure

Davyson et al. (2025) previously published predictors for an antidepressant exposure methylation prediction score (MPS), which was based on self-reported antidepressant exposure in Generation Scotland. These predictors were used to calculate and compare antidepressant exposure MPS between pre-exposure (6-month) and post-exposure (12-month) samples for participants in the SSRI treatment group, using participants who provided both timepoints (18 participants, 36 samples). The MPS was not significantly greater for post-SSRI samples compared to pre-SSRI (t (17) = −1.43, p-value = 0.91; Supplementary Fig. 18). Additionally, the MPS was not associated with clinical severity scores for any of the depression, functioning or psychosis-risk measures tested (Supplementary Fig. 19).

## 4. Discussion

We assessed the effects of 6 months of treatment with the SSRI fluoxetine on the blood-based DNA-methylation and gene expression profiles of a cohort of 60 young people at ultra-high risk for psychosis. We identified 2 CpGs that were significantly associated with SSRI exposure at a multiple-testing corrected significance threshold of p < 0.05, and an additional two at a relaxed threshold of p < 0.1. After 6 months of treatment, all four of these CpGs became hypermethylated in the SSRI group, while methylation levels were either the same or lower in the placebo group relative to before treatment. The top CpG identified, *cg26253898*, was also shown to have a moderate correlation between blood and brain DNA-methylation in BeCON, which may increase the likelihood of this CpG effecting or tagging regulatory changes in response to SSRI exposure in the brain. This CpG annotated to the first intron of the *GCG* gene, which codes for the preproprotein responsible for glucagon, glucagon-like peptide 1 (GLP-1), glucagon-like peptide 2 (GLP-2), oxyntomodulin and glicentin (Drucker, 2003; Kieffer and Habener, 1999). These proteins are involved in the metabolism of glucose and regulation of blood sugar and are expressed in the pancreas, gastrointestinal tract and in the brain, the latter tissue being known to have impaired glucose metabolism in MDD (Videbech, 2000). Additionally, agonists for the receptors of one of these proteins, GLP-1, have recently gained prominence for the treatment of diabetes mellitus and weight loss, and have also demonstrated pro-cognitive and antidepressant effects (Chen et al., 2024; Cooper et al., 2023). These receptor agonists have also demonstrated anti-inflammatory properties and neuroprotective effects, including the protection of the blood–brain barrier (Shan et al., 2019; Lach et al., 2018). The second most significant CpG, *cg09719563*, annotated to *COL23A1*. This gene is associated with anti-inflammatory (M2) macrophages and has previously been shown to become upregulated in the presence of serotonin, largely due to serotonin encouraging the preferential differentiation of M2 macrophages over M1 (pro-inflammatory) macrophages (de las Casas-Engel et al., 2013). *MFGE8*, annotated to *cg26017656*, is likewise another gene involved in M2 macrophage activity, where it enables phagocytosis of apoptotic cells (Spittau et al., 2015). Although M2 macrophages have immune-mediating roles, they also have non-immune functions, such as regulation of neurogenesis and synaptic pruning (Sato, 2015; Birt et al., 2021). Finally, *SGCE*, annotated to *cg22216017*, encodes the epsilon sarcoglycan protein. Two isoforms of this protein, including one that is exclusively expressed in the brain (epsilon-SG2), are enriched in pre- and post-synaptic membranes (Nishiyama et al., 2004) and mouse knock-out models of *Sgce* exhibit major alterations of the serotonergic and dopaminergic systems alongside symptoms of depression and anxiety (Yokoi et al., 2006). Collectively, the known functions of the genes annotated to the significant CpGs support the hypothesis that SSRIs not only have direct effects on serotonin reuptake and release, but also have more widespread effects on inflammation and synaptic remodeling (Walker, 2013; Czeh and Di Benedetto, 2013).

The anti-inflammatory effects of SSRIs are further highlighted by the significant enrichment of genes annotated to the top 100 CpGs in spleen tissue, as the spleen has an important role in the suppression of systemic inflammation (Mota et al., 2019). The spleen produces acetylcholine in response to stimulation by the vagus nerve, which then suppresses the production of pro-inflammatory cytokines (Bassi et al., 2020). In mice, oral consumption of SSRIs has been shown to increase the activity of the vagus nerve and have antidepressant effects that are dependent on this nerve being intact (McVey Neufeld et al., 2019). However, severing this nerve between the stomach and brain prevents the antidepressant effect of oral SSRIs (McVey Neufeld et al., 2019), suggesting that the main pathway for therapeutic effect of oral SSRIs is via the direct stimulation of the brain by the vagus nerve. Therefore, any anti-inflammatory effects of SSRIs that are induced by vagus nerve stimulation of the spleen may be occurring separately to the antidepressant action and may not be a direct contributor to the antidepressant effect. This may explain why SSRI use is associated with a reduction in inflammatory markers, even in the absence of response to treatment (Kohler et al., 2018).

As well as indicating potential mechanisms of action for SSRIs, the results reported here may also provide insight into potential adverse effects. The genes annotated to the significant CpGs are also expressed outside of the brain and have additional functions beyond those previously discussed. For example, proteins encoded by *GCG* and *MFGE8* have gastrointestinal functions, the former including involvement in gastric emptying (Drucker, 2003; Kieffer and Habener, 1999) and the latter in the maintenance of the intestinal mucosa (Raymond et al., 2009). These genes may play a role in the development of gastrointestinal side-effects, including nausea. Meanwhile, mutations in *SGCE* have been linked to the rare movement disorder myoclonus-dystonia (Yokoi et al., 2006; Xiao et al., 2017), while tremor is one of the most commonly reported adverse effects of SSRIs (Friedman, 2020). Additionally, we observed a significant enrichment of SSRI-related genes in adipose and coronary artery tissues, which supports reported side effects of fluoxetine, including incidents of heart disease and reported weight gain. Fluoxetine is known to alter vascular permeability and responsiveness (Rami et al., 2018; Pereira et al., 2017) and increases the risk of atherosclerosis in mice and primates (Rami et al., 2018; Shively et al., 2017), although it still has a lower reported risk for coronary heart disease than other SSRIs in humans (Li et al., 2024). Acute fluoxetine use is known to alter adipocytes by inducing a ‘browning’ of white adipocytes and may preserve leptin sensitivity in obesity (Scabia et al., 2018). Fluoxetine, and other SSRIs, have also been shown to be associated with weight loss in the short term but weight gain in the longer term (>12 months) (Blumenthal et al., 2014; Sussman et al., 2001).

We did not identify any changes in gene expression in response to SSRI treatment, including in the five genes the significant CpGs annotated to. Assuming that these are the most relevant genes for these CpGs, there are several potential reasons for observing significant changes in DNA-methylation but not expression for these genes. Firstly, these genes may display differential splicing (Carvalho and Lasek, 2024) rather than differential expression in response to SSRI exposure, which we would not have been able to identify in our analysis. Secondly, gene expression, and SSRI-induced changes to gene expression, may be more tissue specific than for DNA-methylation (Blake et al., 2020). This tissue-specificity is also relevant for differential splicing, with both *GCG* and *SGCE* known to have tissue-specific isoforms and processing (Nishiyama et al., 2004; Rouille et al., 1997). Alternatively, changes to gene expression may not be as extreme as the changes to DNA-methylation that were detectable in this study. This is supported by a post-hoc power analysis (Supplementary Results), which showed that, despite being a large study of its kind, this study is only powered to detect very large effects and thus was underpowered to detect smaller, biologically plausible SSRI-related changes in both DNA-methylation and gene expression.

We attempted to replicate the signal observed in a previous MWAS of antidepressant use by generating methylation prediction scores (MPS) and analysing its ability to discriminate between the pre- and post-treatment samples in the SSRI group. The MPS performed poorly in our samples, which is likely due to the differences in design between our MWAS and the MWAS used to generate the MPS by Davyson et al. (2025). Chiefly, they used samples taken at a single timepoint and designated cases based on a self-report measure of antidepressant use, which included non-SSRI antidepressants. The length of exposure to the antidepressant also varied within their cohort, which, as they demonstrated themselves, is relevant to the degree of change observed at some CpGs. Additionally, the Generation Scotland cohort is largely comprised of adults in their middle-age (mean age of ~ 47), while our cohort was comprised of young adults and adolescents.

Finally, in addition to analysing DNA-methylation and gene expression changes associated with SSRI exposure, we also examined whether treatment group was associated with changes in clinical scores for depression and UHR-related assessments. We did not identify a significant change in clinical scores associated with treatment with the SSRI or placebo in our cohort. This is in line with the main trial, which did not identify a difference between SSRI and placebo for any of the clinical assessments measured (McGorry et al., 2023). We also did not identify an association between white blood cell proportions or counts and treatment (Supplementary Fig. 20), despite previous reports that SSRIs may reduce the proliferation of some lymphocytes (Martensson and Nassberger, 1993; Diamond et al., 2006; Taler et al., 2007).

The main strength of this analysis is the multi-timepoint, placebo-controlled design. This design allows for a high level of confidence that the associations identified are due to the impact of SSRI exposure and not to any potential confounding demographic or design factors. The main limitation of this analysis, however, is that the sample size, although large for this type of cohort, may nonetheless mean we are not able to detect all associations between SSRI exposure and DNA-methylation or gene expression. This is partially mitigated by the multi-timepoint (repeated measures) design, however replication is warranted to confirm these results if and when other applicable datasets become available. A further limitation is that the STEP cohort, which was explicitly recruited based on young age and enrichment for psychosis risk symptoms, may not reflect broader populations with depression who are likely to be prescribed SSRIs. The focus on SSRI exposure, not response, means that our findings may be less impacted by clinical differences between the STEP cohort and the broader group of SSRI users, however, replication of these findings in adults with MDD or other clinical groups treated with SSRIs is necessary nonetheless to establish the generalisability of these results.

In conclusion, this is the first study (to our knowledge) to explicitly look at DNA-methylation changes in response to SSRI exposure using a randomised clinical trial design. We identify four main CpGs associated with medium-term SSRI use but are not able to identify concurrent changes in gene expression. The identified CpGs, along with the broader pattern of changes in DNA-methylation, highlight the effect of SSRIs on serotonin reuptake, inflammation and glucose metabolism and may provide further insight into potential mechanisms of both the therapeutic actions and adverse effects of SSRIs.

## Supplementary Material

1

2

## Figures and Tables

**Table 1:** Comparison of demographic and clinical characteristics between treatment group for STEP participants who provided 6-month and/or 12-month DNA-methylation samples. Clinical measures taken at 6 months, immediately prior to randomisation to SSRI or Placebo.

Variable	SSRI	Placebo	P-value*	N Missing
Number of Participants^a^	27 (18,3,6)	33 (26,5,2)		
Age	17.7 (2.9)	17.3 (2.5)	0.83	0
% Female	63	58	0.79	0
European ancestry^b^(%)	78	67	0.40	0
Pre-existing psychiatric diagnosis^c^ (%)	85	97	0.31	1
Pre-trial SSRI use^d^ (%)	14.8	12.1	1	0
MADRS total score↑^e^	21.6 (8.9)	19.7 (9.2)	0.51	0
CAARMS total score↑	22.2 (6.6)	16.5 (8.7)	0.021	0
BPRS total score↑	45.8 (7.2)	40.9 (8.6)	0.014	2
SANS total score↓	20.2 (9.8)	16.8 (11.4)	0.13	3
SOFAS score↓	59.0 (11.7)	61.4 (12.3)	0.33	0
DACOBS total score↑	167.1 (32.3)	150.6 (29.7)	0.022	1
Global Functioning: Social↓	6.3 (1.2)	6.3 (1.4)	0.78	0
Global Functioning: Role↓	6.4 (1.6)	6.8 (1.7)	0.18	0

* Bonferroni-adjusted p-value significance threshold is 3.85 × 10^−3^.

a Total number of participants with biological samples (participants with biological samples for both timepoints, participants with 6-month sample only, participants with 12-month sample only).

b ^^^Genetically-inferred ancestry group.

c Comparison of individual diagnostic categories in Supplementary Table 3.

d SSRI use in the 12 months prior to starting the trial.

e Arrow indicates direction of increased impairment, i.e. up-arrows indicate that higher scores mean higher symptom burden while down-arrows indicate that lower scores mean a higher burden of symptoms.

**Table 2:** Methylation sites associated with SSRI exposure at an FDR < 0.1.

CpG	Chr	BP^a^	LogFC	SE	P-value	FDR-adjusted P-value	Gene (SO term^b^)	Relation to UCSC CpG Island	Other traits associated with probe
cg26253898	2	163,006,202	0.025	0.0041	2.79 × 10^−08^*	0.021	GCG (intron)		Gestational age (Spiers et al., 2015), Tissue (Islam et al., 2019), age (Mulder et al., 2021)
cg09719563	5	177,990,438	0.016	0.0028	1.16 × 10^−07^*	0.044	COL23A1 (intron)		
cg22216017	7	94,285,004	0.013	0.0024	3.50 × 10^−07^	0.089	SGCE (intron); PEG10 (2 KB Upstream)	Island	Tissue (Islam et al., 2019), age (Mulder et al., 2021)
cg26017656	15	89,453,368	0.031	0.0057	4.95 × 10^−07^	0.094	MFGE8 (intron)	North Shelf	

* Significant at FDR < 0.05.

a GRCh37 coordinates.

b Sequence Ontology term.

## Data Availability

Data will be made available on request.

## Appendix A. Supplementary data

Supplementary data to this article can be found online at https://doi.org/10.1016/j.bbi.2026.106520.

## References

[R1] Malhi GS, , 2021. The 2020 royal australian and new zealand college of psychiatrists clinical practice guidelines for mood disorders. Aust. N. Z. J. Psychiatry 55, 7–117. 33353391 10.1177/0004867420979353

[R2] Bromet E, , 2011. Cross-national epidemiology of DSM-IV major depressive episode. BMC Med. 9, 90. 21791035 10.1186/1741-7015-9-90 PMC3163615

[R3] Rush AJ, , 2006. Acute and longer-term outcomes in depressed outpatients requiring one or several treatment steps: a STAR*D report. Am. J. Psychiatry 163, 1905–1917. 17074942 10.1176/ajp.2006.163.11.1905

[R4] Lochmann D, Richardson T, 2019. Selective Serotonin Reuptake Inhibitors. Handb. Exp. Pharmacol 250, 135–144. 30838457 10.1007/164_2018_172

[R5] Malhi GS, , 2022. Antidepressant prescribing patterns in Australia. Bjpsych Open 8, e120. 35770420 10.1192/bjo.2022.522 PMC9301763

[R6] Walker FR, 2013. A critical review of the mechanism of action for the selective serotonin reuptake inhibitors: do these drugs possess anti-inflammatory properties and how relevant is this in the treatment of depression? Neuropharmacology 67, 304–317. 23085335 10.1016/j.neuropharm.2012.10.002

[R7] Cipriani A, , 2018. Comparative efficacy and acceptability of 21 antidepressant drugs for the acute treatment of adults with major depressive disorder: a systematic review and network meta-analysis. Lancet 391, 1357–1366. 29477251 10.1016/S0140-6736(17)32802-7 PMC5889788

[R8] Stahl SM, 2000. Blue genes and the monoamine hypothesis of depression. J. Clin. Psychiatry 61, 77–78. 10732653 10.4088/jcp.v61n0201

[R9] Owens MJ, Morgan WN, Plott SJ, Nemeroff CB, 1997. Neurotransmitter receptor and transporter binding profile of antidepressants and their metabolites. J. Pharmacol. Exp. Ther 283, 1305–1322. 9400006

[R10] Hiemke C, Hartter S, 2000. Pharmacokinetics of selective serotonin reuptake inhibitors. Pharmacol. Ther 85, 11–28. 10674711 10.1016/s0163-7258(99)00048-0

[R11] Hindmarch I, 2001. Expanding the horizons of depression: beyond the monoamine hypothesis. Hum. Psychopharmacol 16, 203–218. 12404573 10.1002/hup.288

[R12] Lacasse JR, Leo J, 2005. Serotonin and depression: a disconnect between the advertisements and the scientific literature. PLoS Med. 2, e392. 16268734 10.1371/journal.pmed.0020392 PMC1277931

[R13] Martensson U, Nassberger L, 1993. Influence of antidepressants on mitogen stimulation of human lymphocytes. Toxicol. In Vitro 7, 241–245. 20732195 10.1016/0887-2333(93)90007-r

[R14] Diamond M, Kelly JP, Connor TJ, 2006. Antidepressants suppress production of the Th1 cytokine interferon-gamma, independent of monoamine transporter blockade. Eur. Neuropsychopharmacol 16, 481–490. 16388933 10.1016/j.euroneuro.2005.11.011

[R15] Taler M, , 2007. Immunomodulatory effect of selective serotonin reuptake inhibitors (SSRIs) on human T lymphocyte function and gene expression. Eur. Neuropsychopharmacol 17, 774–780. 17499975 10.1016/j.euroneuro.2007.03.010

[R16] Tynan RJ, , 2012. A comparative examination of the anti-inflammatory effects of SSRI and SNRI antidepressants on LPS stimulated microglia. Brain Behav. Immun 26, 469–479. 22251606 10.1016/j.bbi.2011.12.011

[R17] Czeh B, Di Benedetto B, 2013. Antidepressants act directly on astrocytes: evidences and functional consequences. Eur. Neuropsychopharmacol 23, 171–185. 22609317 10.1016/j.euroneuro.2012.04.017

[R18] Sacre S, Medghalchi M, Gregory B, Brennan F, Williams R, 2010. Fluoxetine and citalopram exhibit potent antiinflammatory activity in human and murine models of rheumatoid arthritis and inhibit toll-like receptors. Arthritis Rheum. 62, 683–693. 20131240 10.1002/art.27304

[R19] Kohler CA, , 2018. Peripheral alterations in cytokine and chemokine levels after antidepressant drug treatment for major depressive disorder: systematic review and meta-analysis. Mol. Neurobiol 55, 4195–4206. 28612257 10.1007/s12035-017-0632-1

[R20] Webb LM, Phillips KE, Ho MC, Veldic M, Blacker CJ, 2020. The relationship between DNA methylation and antidepressant medications: a systematic review. Int. J. Mol. Sci 21, 826. 32012861 10.3390/ijms21030826 PMC7037192

[R21] Moon YK, Kim H, Kim S, Lim SW, Kim DK, 2023. Influence of antidepressant treatment on SLC6A4 methylation in Korean patients with major depression. Am. J. Med. Genet. B Neuropsychiatr. Genet 192, 28–37. 36094099 10.1002/ajmg.b.32921

[R22] Pathak H, Borchert A, Garaali S, Burkert A, Frieling H, 2022. BDNF exon IV promoter methylation and antidepressant action: a complex interplay. Clin. Epigenetics 14, 187. 36572893 10.1186/s13148-022-01415-3 PMC9793565

[R23] Martinowich K, Lu B, 2008. Interaction between BDNF and serotonin: role in mood disorders. Neuropsychopharmacology 33, 73–83. 17882234 10.1038/sj.npp.1301571

[R24] Barbu MC, , 2022. Methylome-wide association study of antidepressant use in Generation Scotland and the Netherlands Twin Register implicates the innate immune system. Mol. Psychiatry 27, 1647–1657. 34880450 10.1038/s41380-021-01412-7 PMC9095457

[R25] Davyson E, , 2025. Insights from a methylome-wide association study of antidepressant exposure. Nat. Commun 16, 1908. 39994233 10.1038/s41467-024-55356-x PMC11850842

[R26] Kallak TK, , 2021. DNA methylation in cord blood in association with prenatal depressive symptoms. Clin. Epigenetics 13, 78. 33845866 10.1186/s13148-021-01054-0 PMC8042709

[R27] Non AL, Binder AM, Kubzansky LD, Michels KB, 2014. Genome-wide DNA methylation in neonates exposed to maternal depression, anxiety, or SSRI medication during pregnancy. Epigenetics 9, 964–972. 24751725 10.4161/epi.28853 PMC4143411

[R28] Lin E, Tsai SJ, 2016. Genome-wide microarray analysis of gene expression profiling in major depression and antidepressant therapy. Prog. Neuropsychopharmacol. Biol. Psychiatry 64, 334–340. 25708651 10.1016/j.pnpbp.2015.02.008

[R29] Menke A, 2013. Gene expression: biomarker of antidepressant therapy? Int. Rev. Psychiatry 25, 579–591. 24151803 10.3109/09540261.2013.825580

[R30] Rayan NA, , 2022. Integrative multi-omics landscape of fluoxetine action across 27 brain regions reveals global increase in energy metabolism and region-specific chromatin remodelling. Mol. Psychiatry 27, 4510–4525. 36056172 10.1038/s41380-022-01725-1 PMC9734063

[R31] Kumar S, , 2019. Transcriptomic changes following chronic administration of selective serotonin reuptake inhibitors: a review of animal studies. Neuropsychopharmacol. Hung 21, 26–35. 30962407

[R32] McGorry PD, , 2023. A sequential adaptive intervention strategy targeting remission and functional recovery in young people at ultrahigh risk of psychosis: the staged treatment in early psychosis (STEP) sequential multiple assignment randomized trial. JAMA Psychiat. 80, 875–885. 10.1001/jamapsychiatry.2023.1947 PMC10308298 37378974

[R33] Hartmann JA, , 2022. Baseline data of a sequential multiple assignment randomized trial (STEP study). Early Interv. Psychiatry10.1111/eip.13263 PMC9795376 35098659

[R34] Nelson B, , 2018. Staged treatment in early psychosis: a sequential multiple assignment randomised trial of interventions for ultra high risk of psychosis patients. Early Interv. Psychiatry 12, 292–306. 28719151 10.1111/eip.12459 PMC6054879

[R35] Barker LF, , 2025. White blood cell proportions are associated with response to psychosocial therapy in young people at ultra-high risk for psychosis. Biol Psychiatry Glob Open Sci 5, 100546. 40697486 10.1016/j.bpsgos.2025.100546 PMC12281354

[R36] Yung AR, , 2005. Mapping the onset of psychosis: the Comprehensive Assessment of At-Risk Mental States. Aust. N. Z. J. Psychiatry 39, 964–971. 16343296 10.1080/j.1440-1614.2005.01714.x

[R37] Maxwell ME Family Interview for Genetic Studies (FIGS): a manual for FIGS. Bethesda, MD: Clinical Neurogenetics Branch, Intramural Research Program, National Institute of Mental Health (1992).

[R38] Montgomery SA, Asberg M, 1979. A new depression scale designed to be sensitive to change. Br. J. Psychiatry 134, 382–389. 444788 10.1192/bjp.134.4.382

[R39] Cornblatt BA, , 2007. Preliminary findings for two new measures of social and role functioning in the prodromal phase of schizophrenia. Schizophr. Bull 33, 688–702. 17440198 10.1093/schbul/sbm029 PMC2526147

[R40] Overall JE, Gorham DR, 1962. The brief psychiatric rating-scale. Psychol. Rep 10, 799–812.

[R41] Andreasen NC, 1989. The scale for the assessment of negative symptoms (SANS): conceptual and theoretical foundations. Br. J. Psychiatry Suppl 49–58. 2695141

[R42] van der Gaag M, , 2013. Development of the davos assessment of cognitive biases scale (DACOBS). Schizophr. Res 144, 63–71. 23332365 10.1016/j.schres.2012.12.010

[R43] Min JL, Hemani G, Davey Smith G, Relton C, Suderman M, 2018. Meffil: efficient normalization and analysis of very large DNA methylation datasets. Bioinformatics 34, 3983–3989. 29931280 10.1093/bioinformatics/bty476 PMC6247925

[R44] Houseman EA, , 2012. DNA methylation arrays as surrogate measures of cell mixture distribution. BMC Bioinf. 13, 86. 10.1186/1471-2105-13-86 PMC3532182 22568884

[R45] Reinius LE, , 2012. Differential DNA methylation in purified human blood cells: implications for cell lineage and studies on disease susceptibility. PLoS One 7, e41361. 22848472 10.1371/journal.pone.0041361 PMC3405143

[R46] Shen X, , 2025. A methylome-wide association study of major depression with out-of-sample case–control classification and trans-ancestry comparison. Nat. Ment. Health10.1038/s44220-025-00486-4 PMC12504109 41069367

[R47] Mulder RH, , 2021. Epigenome-wide change and variation in DNA methylation in childhood: trajectories from birth to late adolescence. Hum. Mol. Genet 30, 119–134. 33450751 10.1093/hmg/ddaa280 PMC8033147

[R48] Tian Y, , 2017. ChAMP: updated methylation analysis pipeline for Illumina BeadChips. Bioinformatics 33, 3982–3984. 28961746 10.1093/bioinformatics/btx513 PMC5860089

[R49] Leek JT, Johnson WE, Parker HS, Jaffe AE, Storey JD, 2012. The sva package for removing batch effects and other unwanted variation in high-throughput experiments. Bioinformatics 28, 882–883. 22257669 10.1093/bioinformatics/bts034 PMC3307112

[R50] Ritchie ME, , 2015. limma powers differential expression analyses for RNA-sequencing and microarray studies. Nucleic Acids Res. 43, e47. 25605792 10.1093/nar/gkv007 PMC4402510

[R51] Battram T, , 2022. The EWAS catalog: a database of epigenome-wide association studies. Wellcome Open Res 7, 41. 35592546 10.12688/wellcomeopenres.17598.1 PMC9096146

[R52] Min JL, , 2021. Genomic and phenotypic insights from an atlas of genetic effects on DNA methylation. Nat. Genet 53, 1311–1321. 34493871 10.1038/s41588-021-00923-x PMC7612069

[R53] Keshawarz A, , 2023. Expression quantitative trait methylation analysis elucidates gene regulatory effects of DNA methylation: the Framingham Heart Study. Sci. Rep 13, 12952. 37563237 10.1038/s41598-023-39936-3 PMC10415314

[R54] Edgar RD, Jones MJ, Meaney MJ, Turecki G, Kobor MS, 2017. BECon: a tool for interpreting DNA methylation findings from blood in the context of brain. Transl. Psychiatry 7, e1187. 28763057 10.1038/tp.2017.171 PMC5611738

[R55] Watanabe K, Taskesen E, van Bochoven A, Posthuma D, 2017. Functional mapping and annotation of genetic associations with FUMA. Nat. Commun 8, 1826. 29184056 10.1038/s41467-017-01261-5 PMC5705698

[R56] Kang HJ, , 2011. Spatio-temporal transcriptome of the human brain. Nature 478, 483–489. 22031440 10.1038/nature10523 PMC3566780

[R57] Consortium, G, 2020. The GTEx Consortium atlas of genetic regulatory effects across human tissues. Science 369, 1318–1330. 32913098 10.1126/science.aaz1776 PMC7737656

[R58] Koopmans F, , 2019. SynGO: an Evidence-based, Expert-Curated Knowledge Base for the Synapse. Neuron 103, 217–234 e4. 31171447 10.1016/j.neuron.2019.05.002 PMC6764089

[R59] Chen Y, Lun AT, Smyth GK, 2016. From reads to genes to pathways: differential expression analysis of RNA-Seq experiments using Rsubread and the edgeR quasi-likelihood pipeline. F1000Res 5, 1438. 27508061 10.12688/f1000research.8987.1 PMC4934518

[R60] Law CW, Chen Y, Shi W, Smyth GK, 2014. voom: Precision weights unlock linear model analysis tools for RNA-seq read counts. Genome Biol. 15, R29. 24485249 10.1186/gb-2014-15-2-r29 PMC4053721

[R61] Spiers H, , 2015. Methylomic trajectories across human fetal brain development. Genome Res. 25, 338–352. 25650246 10.1101/gr.180273.114 PMC4352878

[R62] Islam SA, , 2019. Integration of DNA methylation patterns and genetic variation in human pediatric tissues help inform EWAS design and interpretation. Epigenet. Chrom 12, 1. 10.1186/s13072-018-0245-6 PMC6314079 30602389

[R63] Drucker DJ, 2003. Glucagon-like peptides: regulators of cell proliferation, differentiation, and apoptosis. Mol. Endocrinol 17, 161–171. 12554744 10.1210/me.2002-0306

[R64] Kieffer TJ, Habener JF, 1999. The glucagon-like peptides. Endocr. Rev 20, 876–913. 10605628 10.1210/edrv.20.6.0385

[R65] Videbech P, 2000. PET measurements of brain glucose metabolism and blood flow in major depressive disorder: a critical review. Acta Psychiatr. Scand 101, 11–20. 10674946 10.1034/j.1600-0447.2000.101001011.x

[R66] Chen X, Zhao P, Wang W, Guo L, Pan Q, 2024. The antidepressant effects of GLP-1 receptor agonists: a systematic review and meta-analysis. Am. J. Geriatr. Psychiatry 32, 117–127. 37684186 10.1016/j.jagp.2023.08.010

[R67] Cooper DH, , 2023. Glucagon-like peptide 1 (GLP-1) receptor agonists as a protective factor for incident depression in patients with diabetes mellitus: a systematic review. J. Psychiatr. Res 164, 80–89. 37331261 10.1016/j.jpsychires.2023.05.041

[R68] Shan Y, , 2019. The glucagon-like peptide-1 receptor agonist reduces inflammation and blood-brain barrier breakdown in an astrocyte-dependent manner in experimental stroke. J. Neuroinflamm 16, 242. 10.1186/s12974-019-1638-6 PMC6883580 31779652

[R69] Lach G, Schellekens H, Dinan TG, Cryan JF, 2018. Anxiety, depression, and the microbiome: a role for gut peptides. Neurotherapeutics 15, 36–59. 29134359 10.1007/s13311-017-0585-0 PMC5794698

[R70] de las Casas-Engel M, , 2013. Serotonin skews human macrophage polarization through HTR2B and HTR7. J. Immunol 190, 2301–2310. 23355731 10.4049/jimmunol.1201133

[R71] Spittau B, Rilka J, Steinfath E, Zoller T, Krieglstein K, 2015. TGFbeta1 increases microglia-mediated engulfment of apoptotic cells via upregulation of the milk fat globule-EGF factor 8. Glia 63, 142–153. 25130376 10.1002/glia.22740

[R72] Sato K, 2015. Effects of Microglia on Neurogenesis. Glia 63, 1394–1405. 26010551 10.1002/glia.22858 PMC5032973

[R73] Birt IA, , 2021. Genetic liability for internalizing versus externalizing behavior manifests in the developing and adult hippocampus: insight from a meta-analysis of transcriptional profiling studies in a selectively bred rat model. Biol. Psychiatry 89, 339–355. 32762937 10.1016/j.biopsych.2020.05.024 PMC7704921

[R74] Nishiyama A, Endo T, Takeda S, Imamura M, 2004. Identification and characterization of epsilon-sarcoglycans in the central nervous system. Brain Res. Mol. Brain Res 125, 1–12. 15193417 10.1016/j.molbrainres.2004.01.012

[R75] Yokoi F, Dang MT, Li J, Li Y, 2006. Myoclonus, motor deficits, alterations in emotional responses and monoamine metabolism in epsilon-sarcoglycan deficient mice. J. Biochem 140, 141–146. 16815860 10.1093/jb/mvj138

[R76] Mota CMD, , 2019. Central serotonin prevents hypotension and hypothermia and reduces plasma and spleen cytokine levels during systemic inflammation. Brain Behav. Immun 80, 255–265. 30885841 10.1016/j.bbi.2019.03.017

[R77] Bassi GS, , 2020. Anatomical and clinical implications of vagal modulation of the spleen. Neurosci. Biobehav. Rev 112, 363–373. 32061636 10.1016/j.neubiorev.2020.02.011 PMC7211143

[R78] McVey Neufeld KA, , 2019. Oral selective serotonin reuptake inhibitors activate vagus nerve dependent gut-brain signalling. Sci. Rep 9, 14290. 31582799 10.1038/s41598-019-50807-8 PMC6776512

[R79] Raymond A, Ensslin MA, Shur BD, 2009. SED1/MFG-E8: a bi-motif protein that orchestrates diverse cellular interactions. J. Cell. Biochem 106, 957–966. 19204935 10.1002/jcb.22076 PMC2742659

[R80] Xiao J, , 2017. Role of major and brain-specific Sgce isoforms in the pathogenesis of myoclonus-dystonia syndrome. Neurobiol. Dis 98, 52–65. 27890709 10.1016/j.nbd.2016.11.003 PMC5283163

[R81] Friedman JH, 2020. Movement disorders induced by psychiatric drugs that do not block dopamine receptors. Parkinsonism Relat. Disord 79, 60–64. 32871538 10.1016/j.parkreldis.2020.08.031

[R82] Rami M, , 2018. Chronic Intake of the Selective Serotonin Reuptake Inhibitor Fluoxetine Enhances Atherosclerosis. Arterioscler. Thromb. Vasc. Biol 38, 1007–1019. 29567680 10.1161/ATVBAHA.117.310536

[R83] Pereira CA, , 2017. Chronic treatment with fluoxetine modulates vascular adrenergic responses by inhibition of pre- and post-synaptic mechanisms. Eur. J. Pharmacol 800, 70–80. 28216049 10.1016/j.ejphar.2017.02.029

[R84] Shively CA, Silverstein-Metzler M, Justice J, Willard SL, 2017. The impact of treatment with selective serotonin reuptake inhibitors on primate cardiovascular disease, behavior, and neuroanatomy. Neurosci. Biobehav. Rev 74, 433–443. 27590831 10.1016/j.neubiorev.2016.08.037 PMC5366071

[R85] Li FL, , 2024. The association between the fluoxetine use and the occurrence of coronary heart disease: a nationwide retrospective cohort study. BMC Cardiovasc. Disord 24, 628. 39522052 10.1186/s12872-024-04280-5 PMC11549814

[R86] Scabia G, , 2018. The antidepressant fluoxetine acts on energy balance and leptin sensitivity via BDNF. Sci. Rep 8, 1781. 29379096 10.1038/s41598-018-19886-x PMC5789051

[R87] Blumenthal SR, , 2014. An electronic health records study of long-term weight gain following antidepressant use. JAMA Psychiat. 71, 889–896. 10.1001/jamapsychiatry.2014.414 PMC9980723 24898363

[R88] Sussman N, Ginsberg DL, Bikoff J, 2001. Effects of nefazodone on body weight: a pooled analysis of selective serotonin reuptake inhibitor- and imipramine-controlled trials. J. Clin. Psychiatry 62, 256–260. 11379839

[R89] Carvalho L, Lasek AW, 2024. It is not just about transcription: involvement of brain RNA splicing in substance use disorders. J. Neural Transm. (Vienna) 131, 495–503. 38396082 10.1007/s00702-024-02740-y PMC11055753

[R90] Blake LE, , 2020. A comparison of gene expression and DNA methylation patterns across tissues and species. Genome Res. 30, 250–262. 31953346 10.1101/gr.254904.119 PMC7050529

[R91] Rouille Y, Bianchi M, Irminger JC, Halban PA, 1997. Role of the prohormone convertase PC2 in the processing of proglucagon to glucagon. FEBS Lett. 413, 119–123. 9287128 10.1016/s0014-5793(97)00892-2

