# Supplementary material for: Effects of 6-months of SSRI use on DNA-methylation and gene expression in blood
Source: Brain Behav Immun. Author manuscript; Available in PMC 2026 Jun 29. (PMC13313880; doi:10.1016/j.bbi.2026.106520)
Supplement: 2 [file NIHMS2189092-supplement-2.docx]

Table of Contents

[STEP trial design 2](#_Toc214027481)

[Supplementary Methods 3](#_Toc214027482)

[Genome-wide genotyping 3](#_Toc214027483)

[Preparation of DNA Methylation samples 3](#_Toc214027484)

[QC of DNA Methylation data 4](#_Toc214027485)

[RNA sequencing 5](#_Toc214027486)

[RNA Mapping and QC 5](#_Toc214027487)

[Supplementary Results 5](#_Toc214027488)

[MWAS 5](#_Toc214027489)

[MWAS Sensitivity Analysis: Age 10](#_Toc214027490)

[MWAS Sensitivity Analysis: BMI 11](#_Toc214027491)

[MWAS Follow-up 13](#_Toc214027492)

[Correlation of DNA-methylation between SSRI Exposure and MDD 13](#_Toc214027493)

[BeCON correlation between blood and brain tissues 14](#_Toc214027494)

[Enrichment of genes annotated to top 100 CpGs 16](#_Toc214027495)

[Differential Expression 19](#_Toc214027496)

[Antidepressant Methylation Profile Score 22](#_Toc214027497)

[Association of Antidepressant MPS with clinical scores 23](#_Toc214027498)

[Association of white blood cells with SSRI Exposure 25](#_Toc214027499)

[Post-hoc power analysis 27](#_Toc214027500)

[MWAS G*Power results: 27](#_Toc214027501)

[Differential Expression G*Power results: 27](#_Toc214027502)

[Formulas used 29](#_Toc214027503)

[Univariate testing of demographic and clinical variables on Treatment group 29](#_Toc214027504)

[Association of clinical measures with SSRI Exposure 29](#_Toc214027505)

[Association of white blood cells with SSRI Exposure 29](#_Toc214027506)

[MWAS 29](#_Toc214027507)

[Differential Expression 29](#_Toc214027508)

[Antidepressant MPS 29](#_Toc214027509)

[References 30](#_Toc214027510)

# STEP trial design


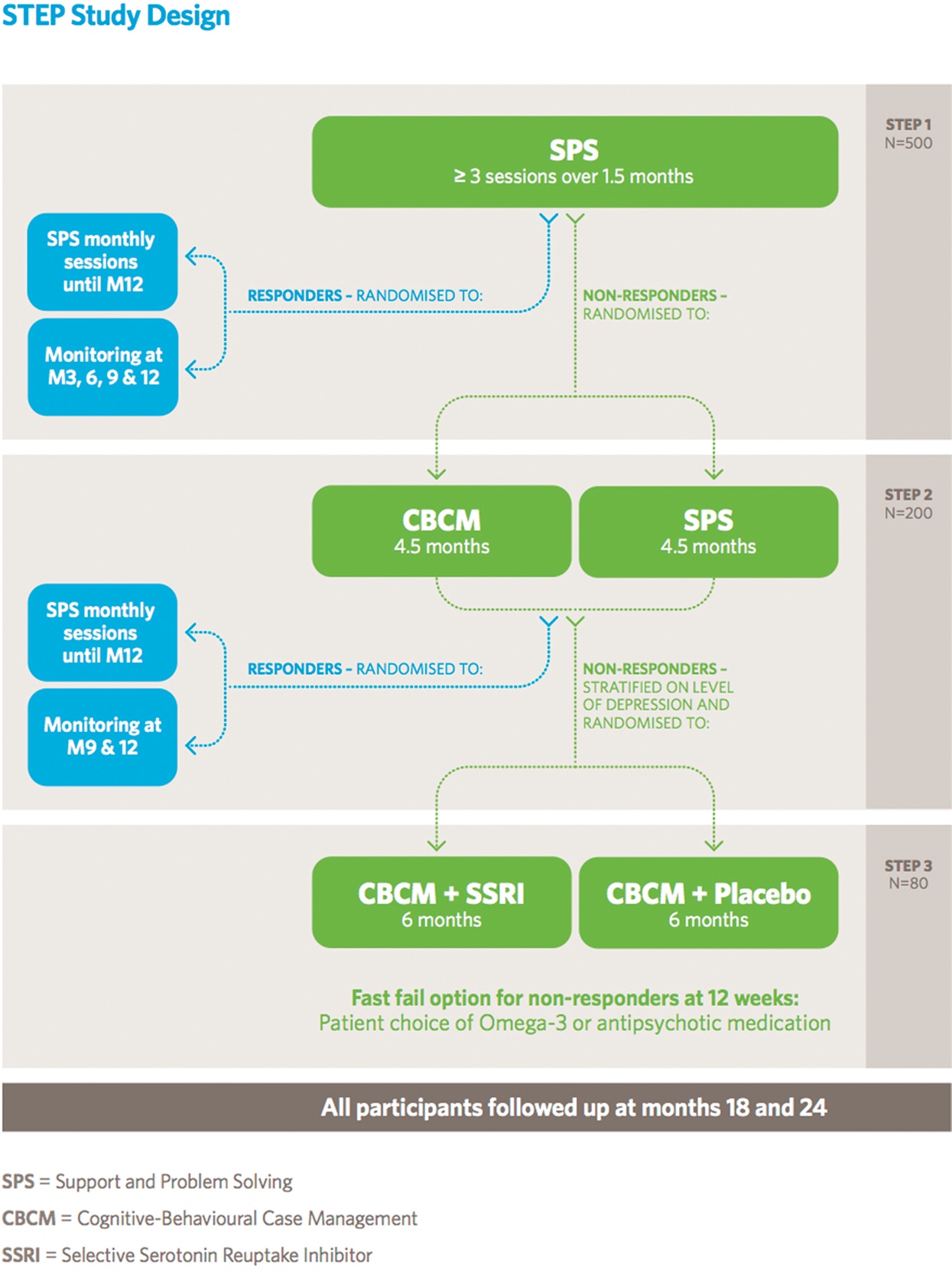


Supplementary Figure 1. Staged Treatment in Early Psychosis (STEP) study design (https://doi.org/10.1111/eip.12459)

# Supplementary Methods

## Genome-wide genotyping

Genotyping was performed using the Illumina Infinium Global Screening Array (GSAMD) on DNA derived from either saliva (n=193) or whole blood (n=15). Initial quality control was performed per batch using PLINK v1.9^1^. Samples were removed if they had high missingness (>10% of SNPs). SNPs were removed if they had high missingness (>10% of samples), minor allele frequency (MAF) less than 0.01, or deviated from Hardy-Weinberg equilibrium (p-value < 1x10^-6^). Samples were imputed to the Haplotype reference consortium (HRC) (Version r1.1 2016). Imputed SNPs were removed if they had an imputation INFO score less than 0.8, MAF less than 0.01 or deviated from Hardy-Weinberg equilibrium. Genetic sex was imputed using PLINK and compared to participant’s reported gender. Participants were assigned to broad ancestry groups based on principal component analysis of genetic similarity to predefined sets of individuals from the 1000 genomes project^2^. Genetically-inferred ancestry was compared to reported countries of birth for participants and their parents, where available. Genetic relatedness of participants was estimated for all samples, as well as within genetic-ancestry groups, using GCTA^3^. Duplicated samples (identified using genetic relatedness) were removed, as was one member of a pair of confirmed full-siblings. The sibling with less missing data was retained.

## Preparation of DNA Methylation samples

DNA concentration was quantified using the Take3™ Micro-Volume Plate on the Epoch™ Microplate Spectrophotometer (BioTek Instruments, Inc.) and samples with low concentration ( < 25 ng/µl) were re-extracted. Bisulfite conversion was then performed with the Zymo EZ-96 DNA Methylation™ Kit Zymo Research, Irvine, CA, USA) and samples with enough converted DNA concentrations (>19 ng/µl) were taken forward to be assayed.

12 samples were excluded from the methylation assay. One sample was excluded due to failing bisulphite conversion and there was insufficient sample left for a re-run. All other exclusions were of duplicate samples, as only one of the duplicates was required.

Methylation levels were measured using the Illumina Infinium® MethylationEPIC BeadChip Assay. The samples were run in three batches of 96 samples, with the remaining samples run across two partial plates with samples from another study. Sample location on the plates was randomised, and human DNA controls and plate replicates were included.

Illumina Genome Studio v1.0 software was used to check the quality of the methylation assay output. All but two samples passed at a threshold of >860 000 site captured, and the majority of samples were very high quality.

## QC of DNA Methylation data

QC and normalisation were performed with the R package Meffil, using recommended parameters^4^. Samples were removed if they had dye bias, low methylation quality, sex mismatches, or had failed any of the pre-sequencing checks. Two samples belonging to a participant with known Klinefelter’s syndrome (i.e. sex chromosome complement XXY) were identified, but not removed, when checking for sex outliers. Methylation probes were removed if they had poor detection scores. Probes displaying cross-reactivity or overlap with SNPs or repetitive elements were also removed, as recommended elsewhere^5^. After QC, 351 samples and 779,503 probes remained.

Given the longitudinal study design, each participant may have contributed multiple samples. Methylation samples were, therefore, checked for identity against each other and against participant genotypes using the 59 SNPs on the EPIC array. Two mislabelled samples were identified and updated to reflect their correct origin, which was ascertained by genotype and other DNA-methylation sample matches from the true identities.

## RNA sequencing

Whole blood was collected in PAXgene tubes and extracted using the PAXgene Blood RNA kit (Qiagen). 5ul aliquots of each sample were used to assess RNA concentration and integrity with Agilent’s EPOCH and Bioanalyzer. Samples were prepared for sequencing using Stranded Total RNA Ribo-zero plus prep kits (Illumina) followed by Paired End sequencing on Novaseq 6000 S4 v1.5 2x100bp flowcells (Illumina).

## RNA Mapping and QC

Initial processing of sequenced reads was performed using fastp^6^, which removes empty reads and trims any adapters detected on the reads. Reads were mapped to the GENCODE human genome primary assembly (v38) using STAR^7^. The non-default ENCODE mapping parameters were used and two-pass mapping was performed, in order to better account for reads spanning unannotated splice junctions. Mapping quality was high for all samples (>80% uniquely mapping reads). Reads were then quantified using Rsubread’s featureCounts^8^, requiring both ends of a fragment to be mapped.

RNA samples were checked for identity against each other and against participant genotypes using SNVs called via GATK4 v4.2.1.0’s ‘RNAseq short variant discovery (SNPs + Indels)’ best practices workflow^9^.

# Supplementary Results

## MWAS

MWAS were initially performed using only participants that had eligible samples for both timepoints, however, to maximise power, a subsequent MWAS was performed that also included participants who provided a sample for only one of the two timepoints. Probes with a Benjamini-Hochberg-adjusted p-value less than 0.1 were considered significant.

Log-fold changes for both the significant CpGs and all tested CpGs were highly correlated between the strictly repeated-measures MWAS and the MWAS with all eligible samples, indicating the inclusion of participants with only one timepoint had little effect on the results (R = 0.87, p-value < 2.2x10^-16^ ; Supplementary Figure 2 & Supplementary Figure 3).


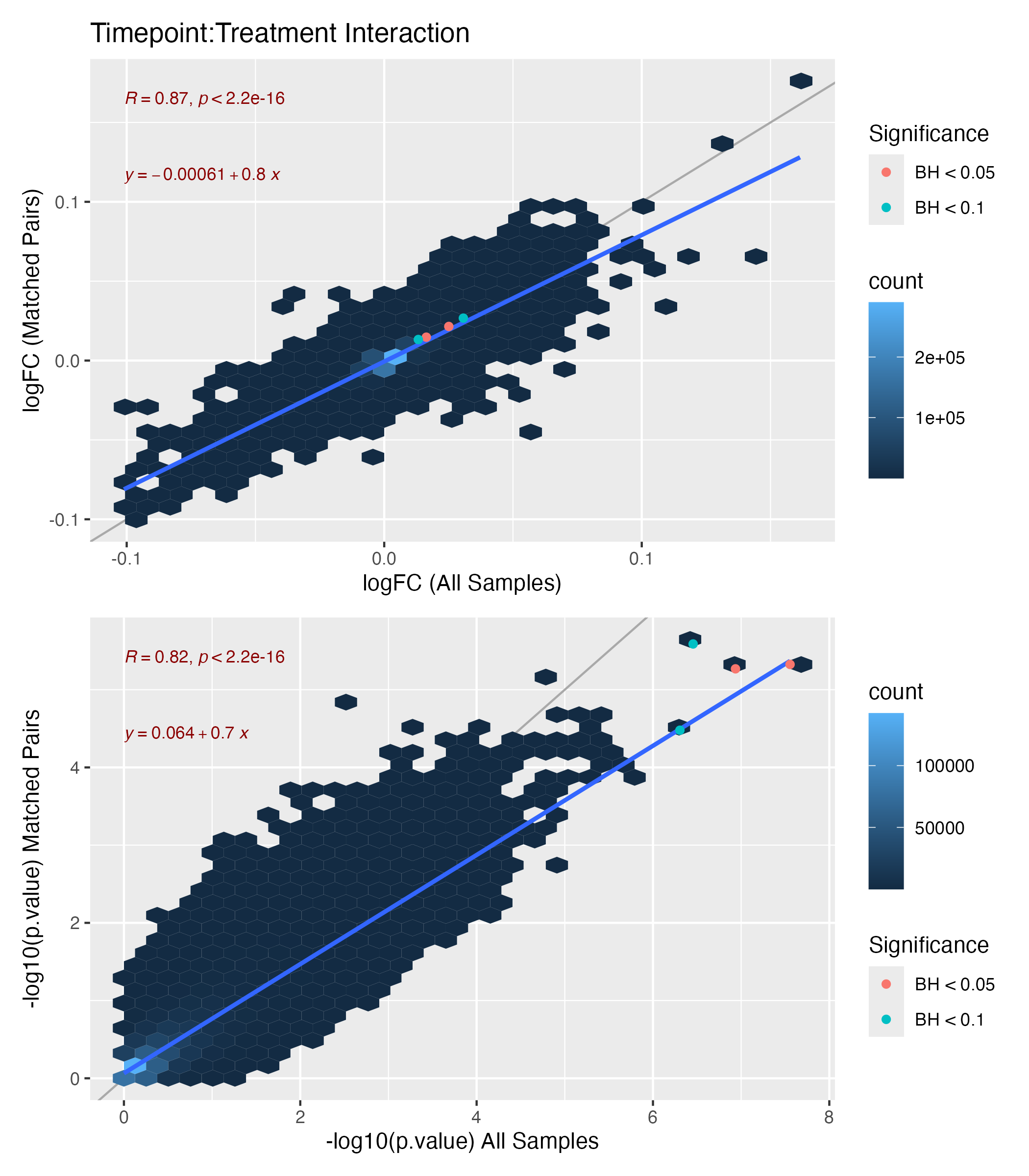


Supplementary Figure 2. Results for MWAS on SSRI exposure (interaction between timepoint and treatment group) for the matched-pairs sample subset vs All eligible samples. Matched Pairs refers to participants with both a 6-month and 12-month DNA-methylation sample, while All Samples refers to all participants with a 6-month OR 12-month sample who meet inclusion criteria. Significance refers to Benjamini-Hochberg adjusted p-values for the All Samples MWAS. Probes are marked in red if they met an adjusted p-value threshold of 0.05 and coloured in teal If they met a threshold of 0.1.


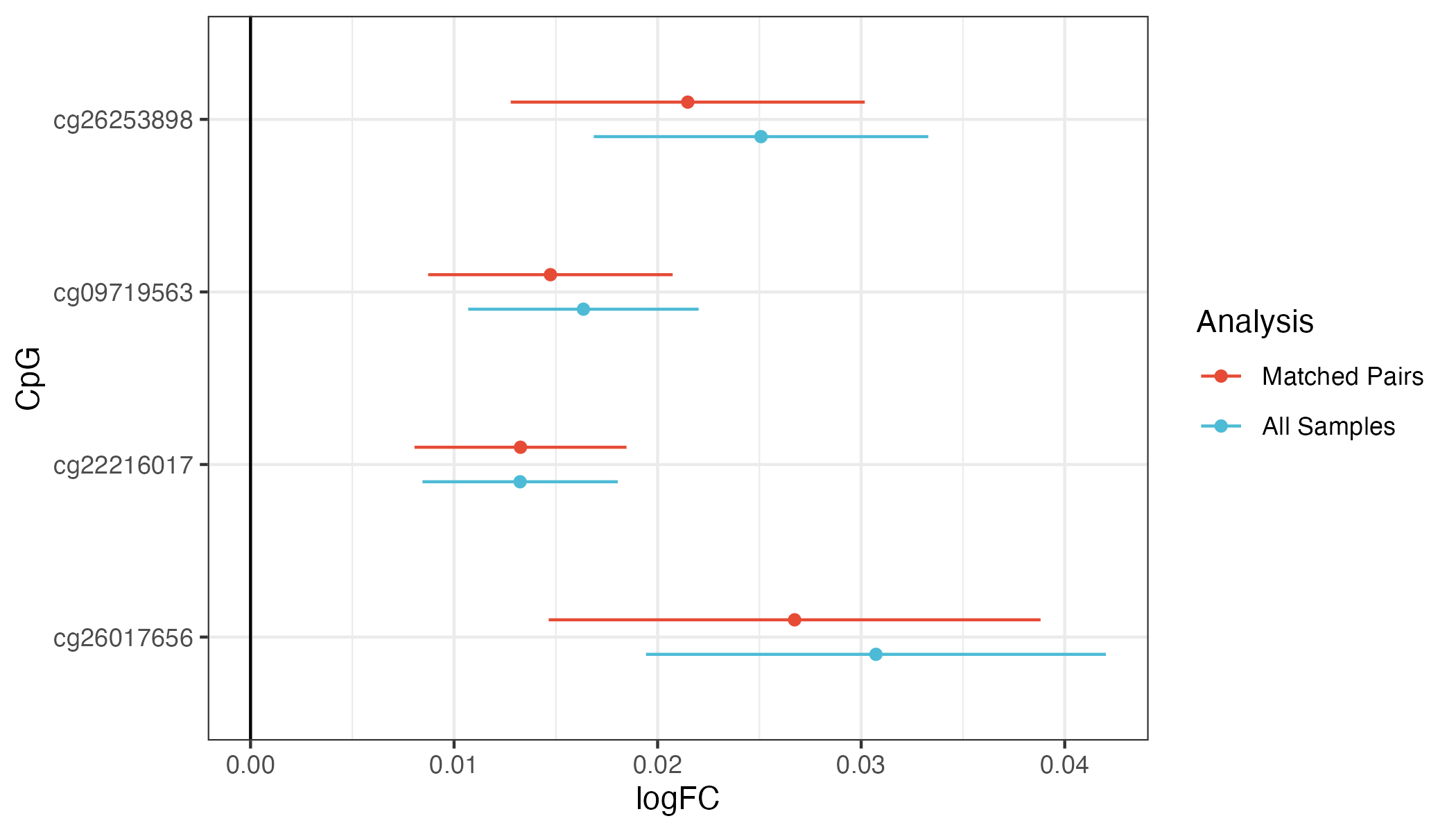


Supplementary Figure 3. Comparison of logFC and 95% CI for the 4 significant CpGs between matched pairs and all-samples.


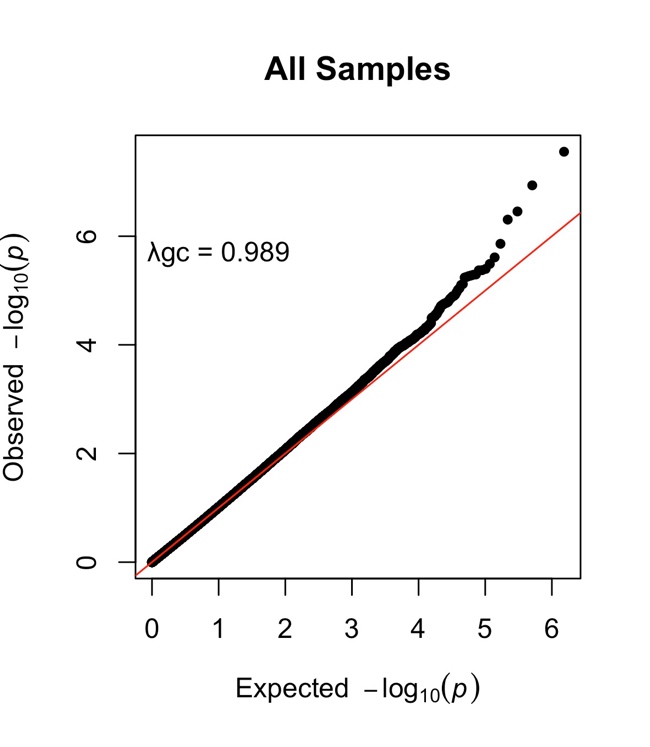

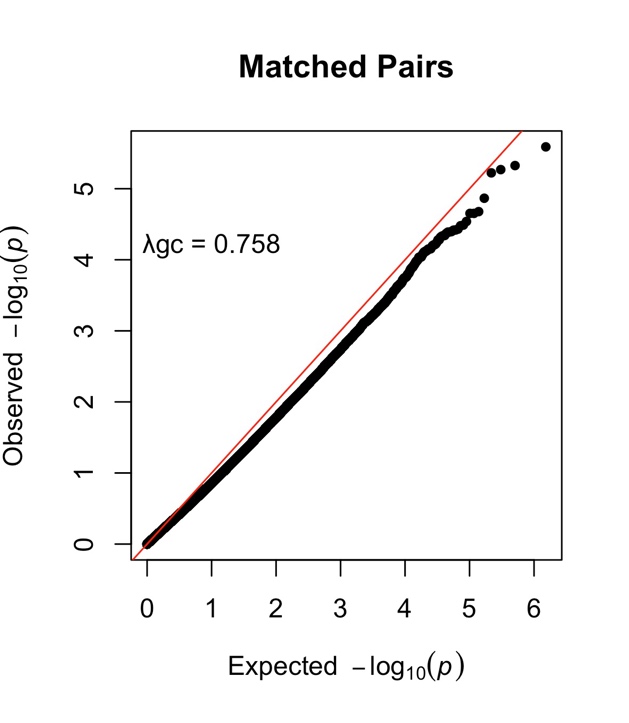


A)

B)

Supplementary Figure 4. QQ-plots for the timepoint:treatment interaction in the MWAS for A) the matched-pairs sample subset and B) All eligible samples. λgc is the genomic inflation factor.


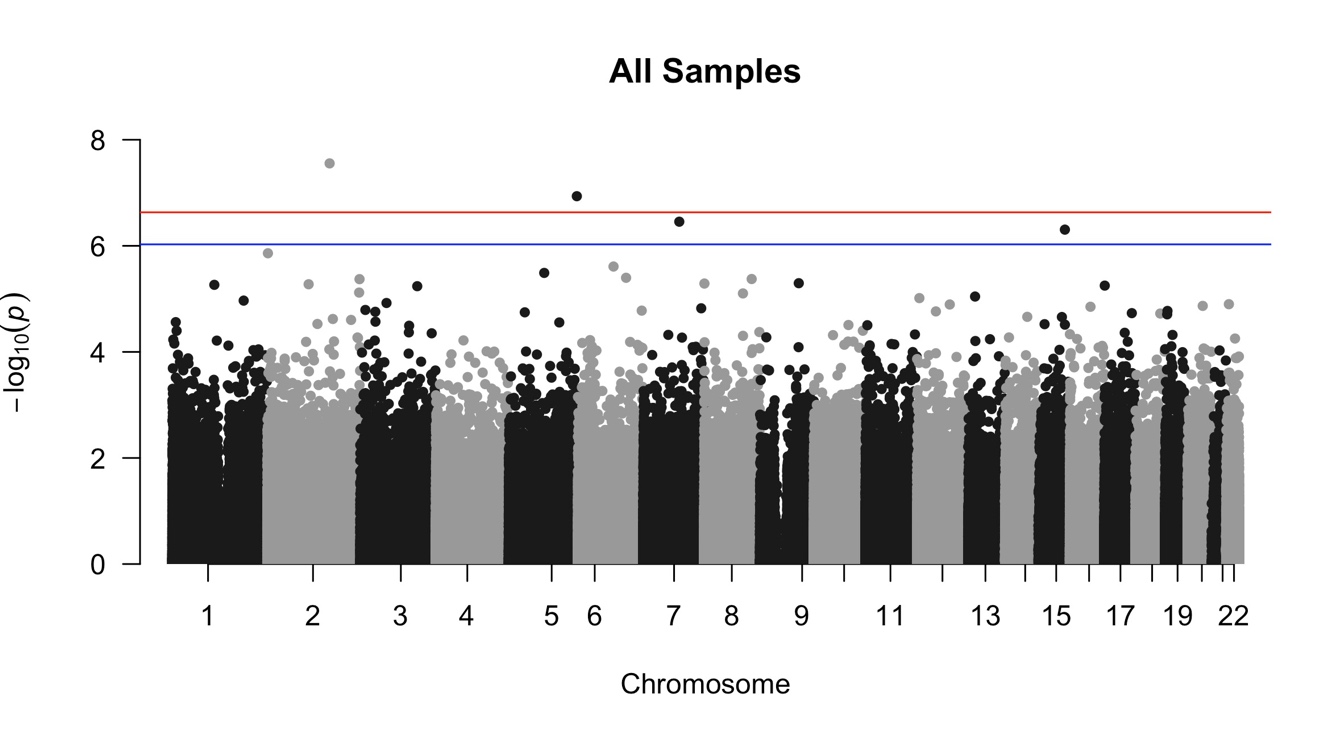

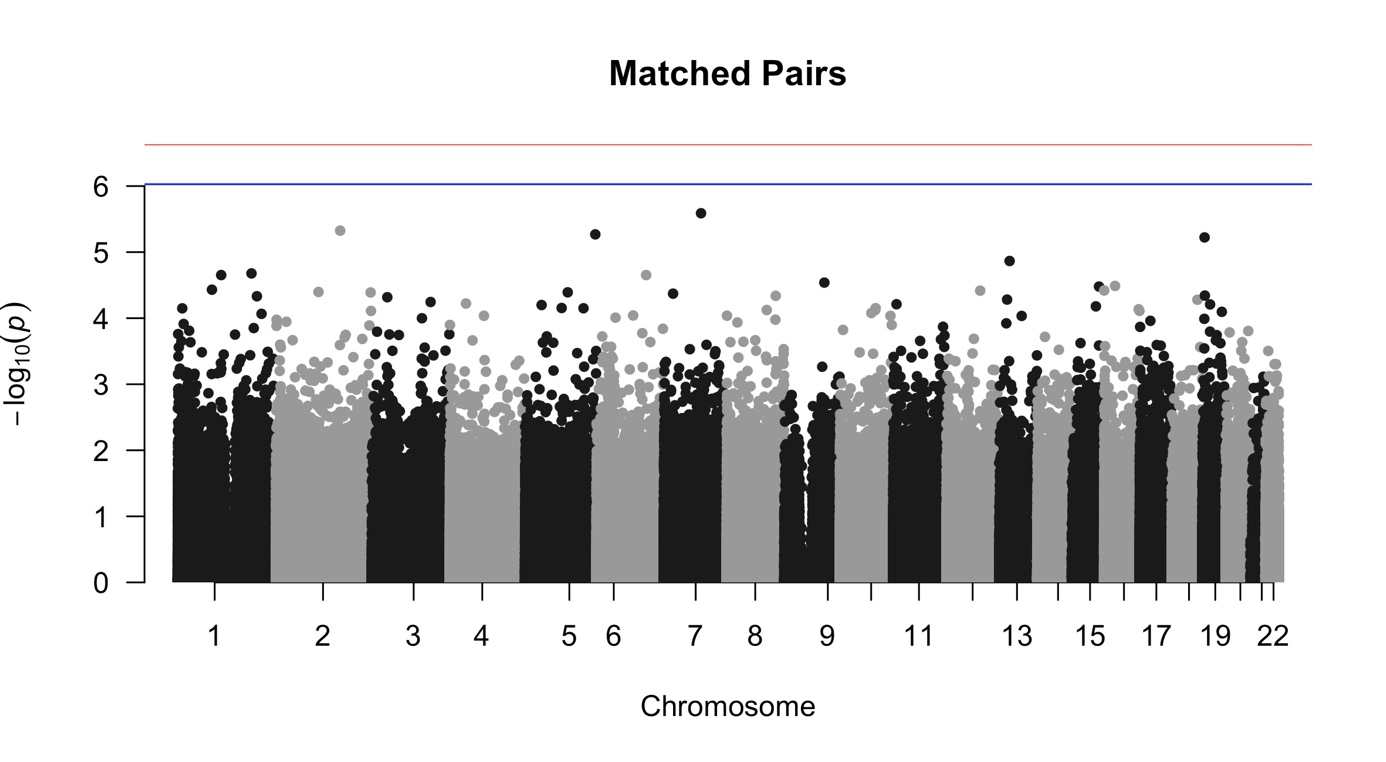


A)

B)

Supplementary Figure 5. MWAS associations with the timepoint:treatment interaction for A) the matched-pairs sample subset and B) All eligible samples. The significance thresholds shown here are approximate, as significance was determined using the Benjamini-Hochberg (BH) procedure on the obtained p-values. As no CpGs achieved a p_BH_ < 0.1 in the Matched-Pairs analysis, the red line on both plots signifies the approximate maximum p-value that would result in a p_BH_ < 0.05 in the All-samples analysis, while the blue line on both plots signifies the approximate maximum p-value that would result in a p_BH_ < 0.1 in the All-samples analysis.

## MWAS Sensitivity Analysis: Age

DNA-methylation associations with age from the STEP MWAS were compared to those from the Epidelta study of DNA-methylation during childhood and adolescence^10^ , in order to assess whether age-related trends were consistent with the latter study. DNA-methylation probe associations with Epidelta’s Model 1 (age as a fixed effect) were downloaded from the MRCIEU EWAS catalog. This Epidelta model was chosen as it best reflected how age was modelled in the STEP SSRI MWAS.

The correlation between the Epidelta beta and the STEP log-fold-change for age was calculated for three sets of probes: a) All probes that were present in both datasets, b) the most significantly associated Epidelta probes (p < 1 x 10^-16^, recorded in the EWAS catalog as p=0E+00), and c) the top 500 most significant probes for age in the STEP SSRI MWAS. For the latter two comparisons, the threshold was first applied to the relevant dataset and then the correlation was calculated using the subset of probes present in both datasets.

There was a significant correlation between the Epidelta beta and STEP log-fold-change when all probes present in both datasets were included (Supplementary Figure 6). This correlation increased when probes were subset to the most significant in the Epidelta MWAS (R=0.28) or the STEP MWAS (R=0.63).


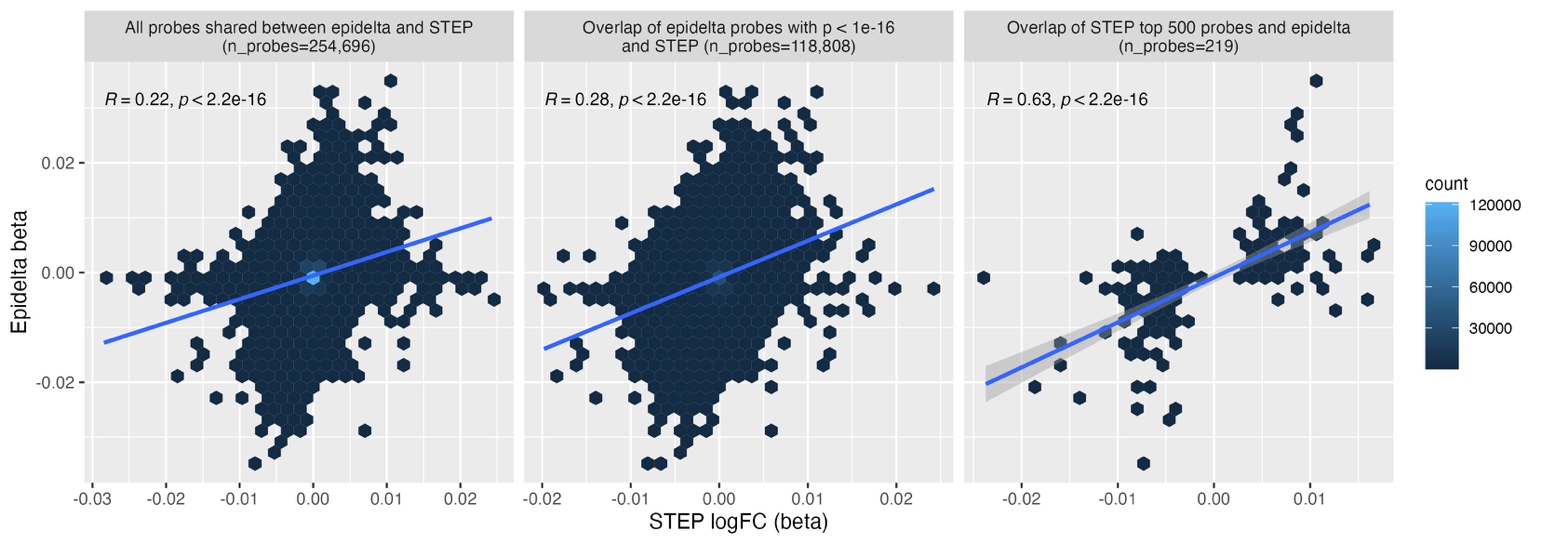


Supplementary Figure 6. Correlation between Epidelta and STEP MWAS betas for age. A) All probes present in both datasets. B) Thresholding by the most significantly associated Epidelta probes. C) Thresholding by the most 500 most significant STEP probes for age. Note: LogFC is used for consistency with other results in this paper, however these estimates are interpretable similarly to beta coefficients when using a quantitative trait such as age, as they represent the change in methylation associated with a one-unit increase in the variable)

## MWAS Sensitivity Analysis: BMI

We observed a significant enrichment of genes annotated to the top 100 CpGs in adipose tissue. To ensure this was not due to potential confounding with BMI, we assessed whether the association of the top 100 CpGs with the Timepoint:Treatment interaction was altered by the inclusion of BMI. BMI was not initially included as a covariate due to high levels of missingness (>10%) for participants’ height and weight measurements, which were used to calculate BMI with the standard formula of kg/m^2^.

Samples with missing BMI measures were removed and limma was used to perform an ‘MWAS’ on ComBAT-corrected betas for the top 100 CpGs from the main MWAS. An initial ‘MWAS’ was performed using the same model as the main SSRI exposure MWAS, to account for changes in results due to the removal of samples with missing BMI measurements, followed by a second ‘MWAS’ that included BMI as an additional covariate. The effect of including BMI on the association of the Timepoint:Treatment interaction was then assessed using the correlation between estimated coefficients of the original model compared to the BMI-inclusion model (Supplementary Figure 7).

Including BMI as a covariate does not meaningfully affect the association between SSRI exposure (modelled as the interaction between timepoint and treatment) and the methylation values of the top 100 CpGs from the main MWAS.


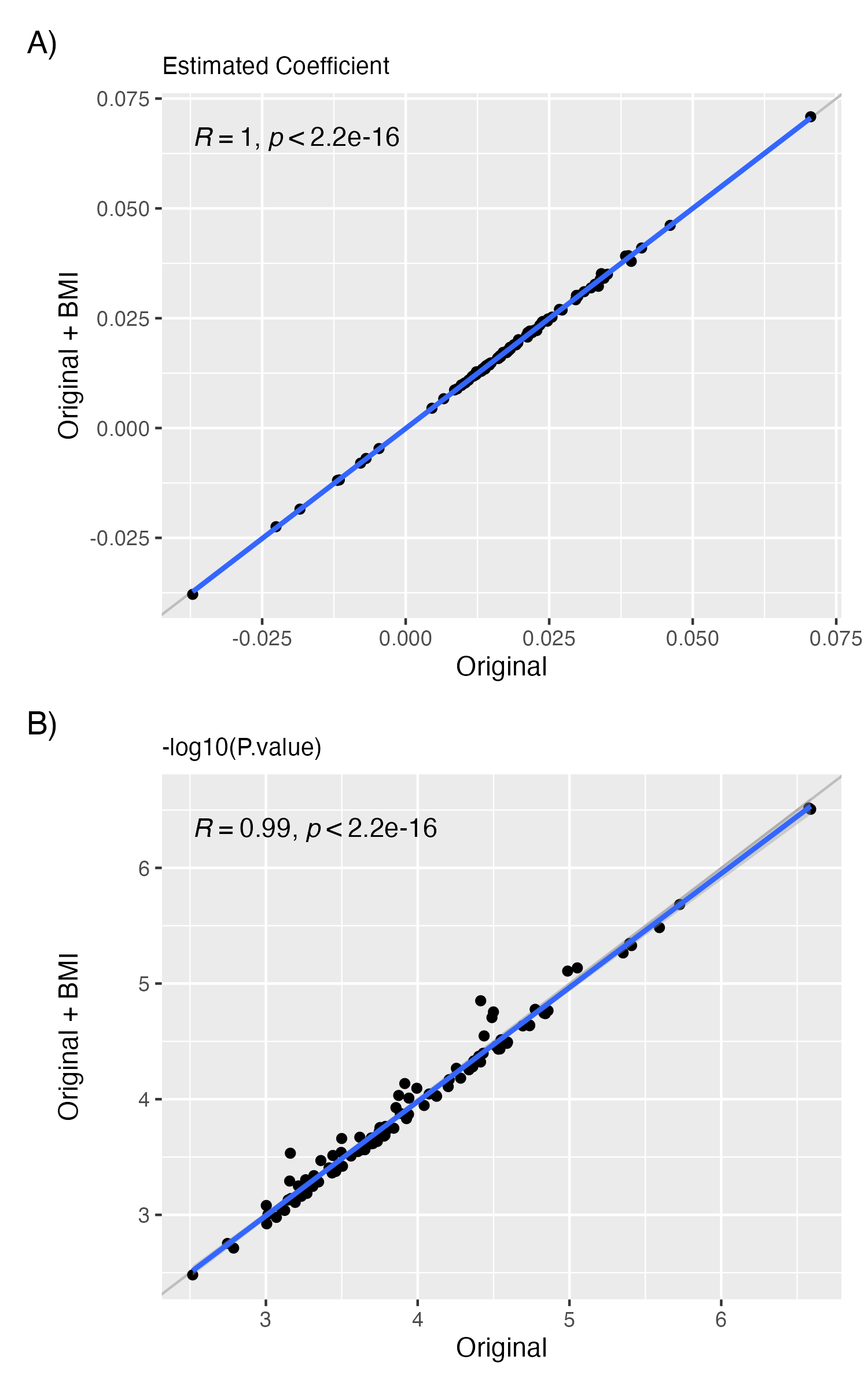


Supplementary Figure 7. The estimated coefficients (A) and P-values (B) of the association of the top 100 CpGs with SSRI exposure (Timepoint:Treatment Interaction). ‘Original’ refers to the association analysis performed using the same model as the main MWAS. ‘Original + BMI’ refers to the association analysis performed using the same model plus the addition of BMI. Samples with missing BMI measures have been excluded from both analyses, which means the Original model had to be re-run in this subset of samples in order to be a valid comparison to the BMI model.

## MWAS Follow-up

Correlation of DNA-methylation between SSRI Exposure and MDD


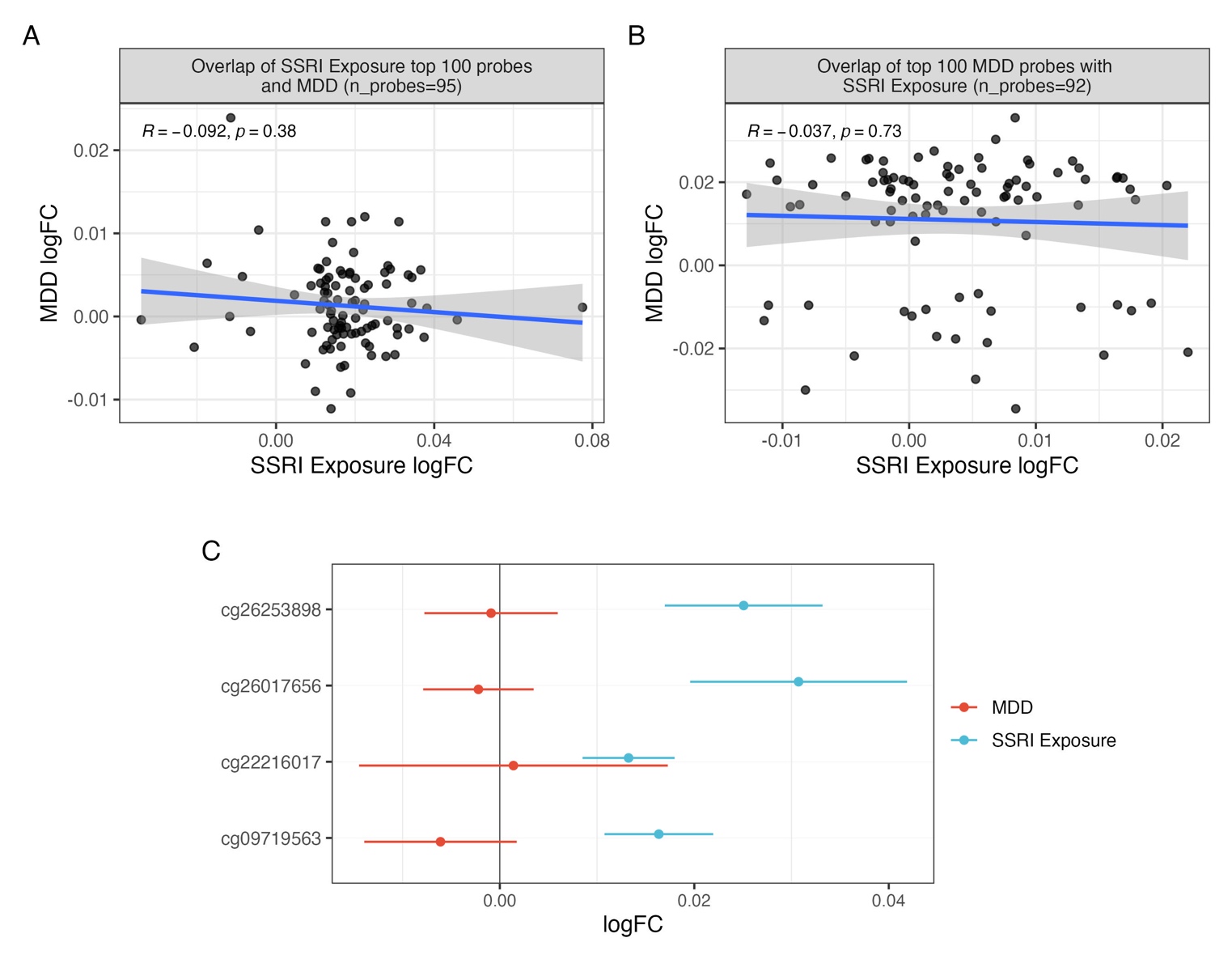


Supplementary Figure 8. Comparison of MWAS associations between SSRI exposure and MDD. A) Thresholding by the top 100 most significant SSRI Exposure probes. B) Thresholding by the top 100 most significant MDD probes. C) The log-fold-change and 95% CI of association with MDD and SSRI exposure for the 4 significant SSRI exposure probes.

In order to examine whether CpGs implicated in SSRI exposure show associations with major depressive disorder (MDD), top CpGs from our SSRI exposure MWAS were compared against the results of a recently published, large meta-analysis of DNA-methylation associations with lifetime diagnosis of major depression^11^.

None of the four significant SSRI exposure probes were also significant for MDD and nor were any of the 15 probes with p_Bonferroni_ < 0.05 for MDD significant, or approaching significant, for SSRI exposure (Supplementary Figure 8.C).

Reasoning that any relationship between the two phenotypes would be most observable in the most strongly associated probes, we compared the logFC of the top 100 probes for SSRI exposure against their logFC in the MDD MWAS (Supplementary Figure 8.A), and the logFC of the top 100 probes for MDD against their logFC for SSRI exposure (Supplementary Figure 8.B). There were no probes that were in the top 100 of both traits, and no significant correlations were observed between logFCs of the two traits.

BeCON correlation between blood and brain tissues

Supplementary Figure 9. BeCON comethylation plots for cg26253898 and cg22216017. The other two significant probes (cg09719563, cg26017656) were not available in the BeCON comethylation database.

Supplementary Figure 10. BeCON comethylation summary tables for cg26253898 and cg22216017. The other two significant probes (cg09719563, cg26017656) were not available in the BeCON comethylation database.

Enrichment of genes annotated to top 100 CpGs


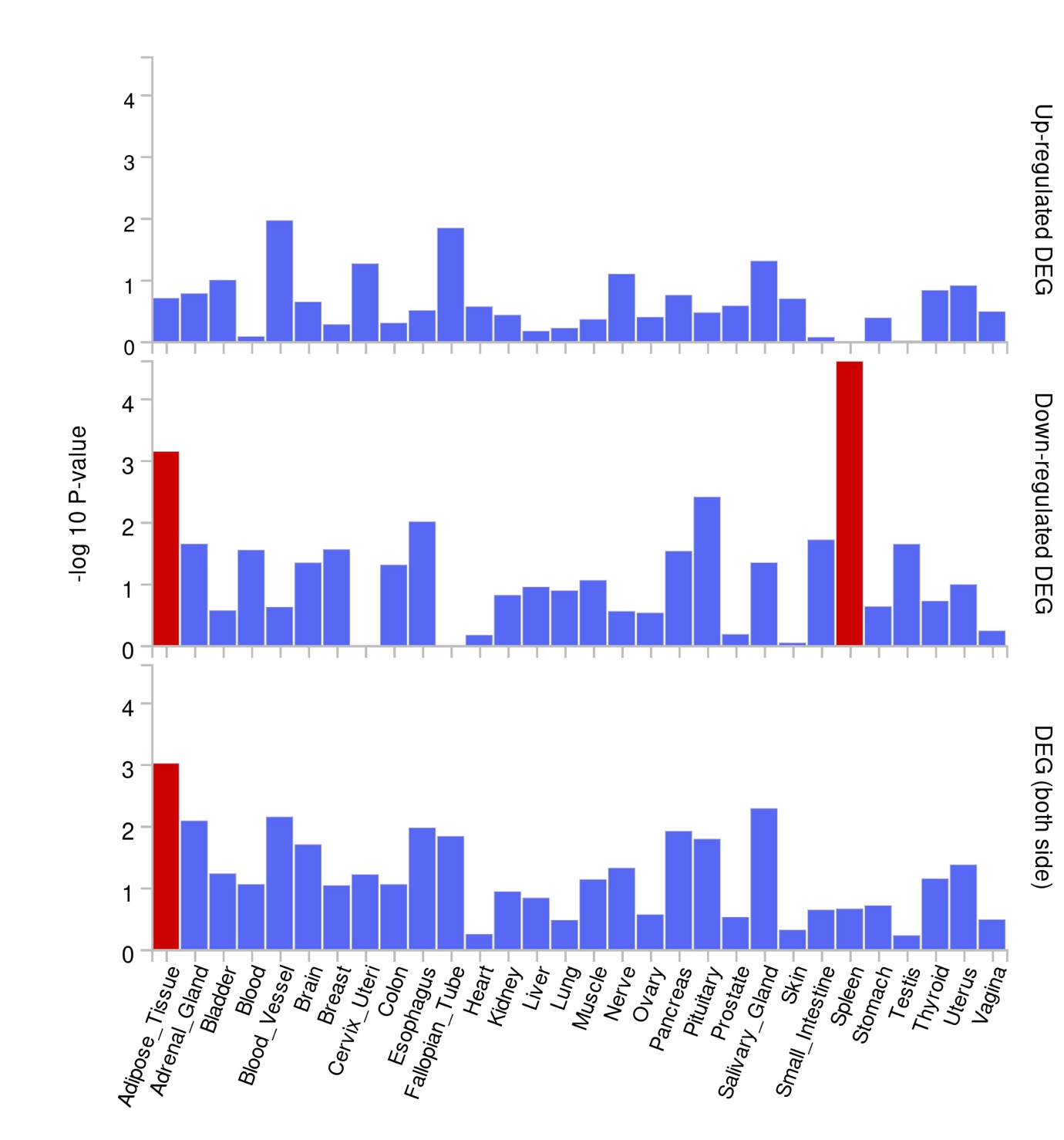


Supplementary Figure 11. Results of gene-set enrichment analysis in GTEX v8 30 general tissue type dataset for genes annotated to the top 100 CpGs associated with SSRI exposure. Gene-set enrichment performed using FUMA GENE2FUNC. Bonferroni-adjusted p-values < 0.05 considered significant.


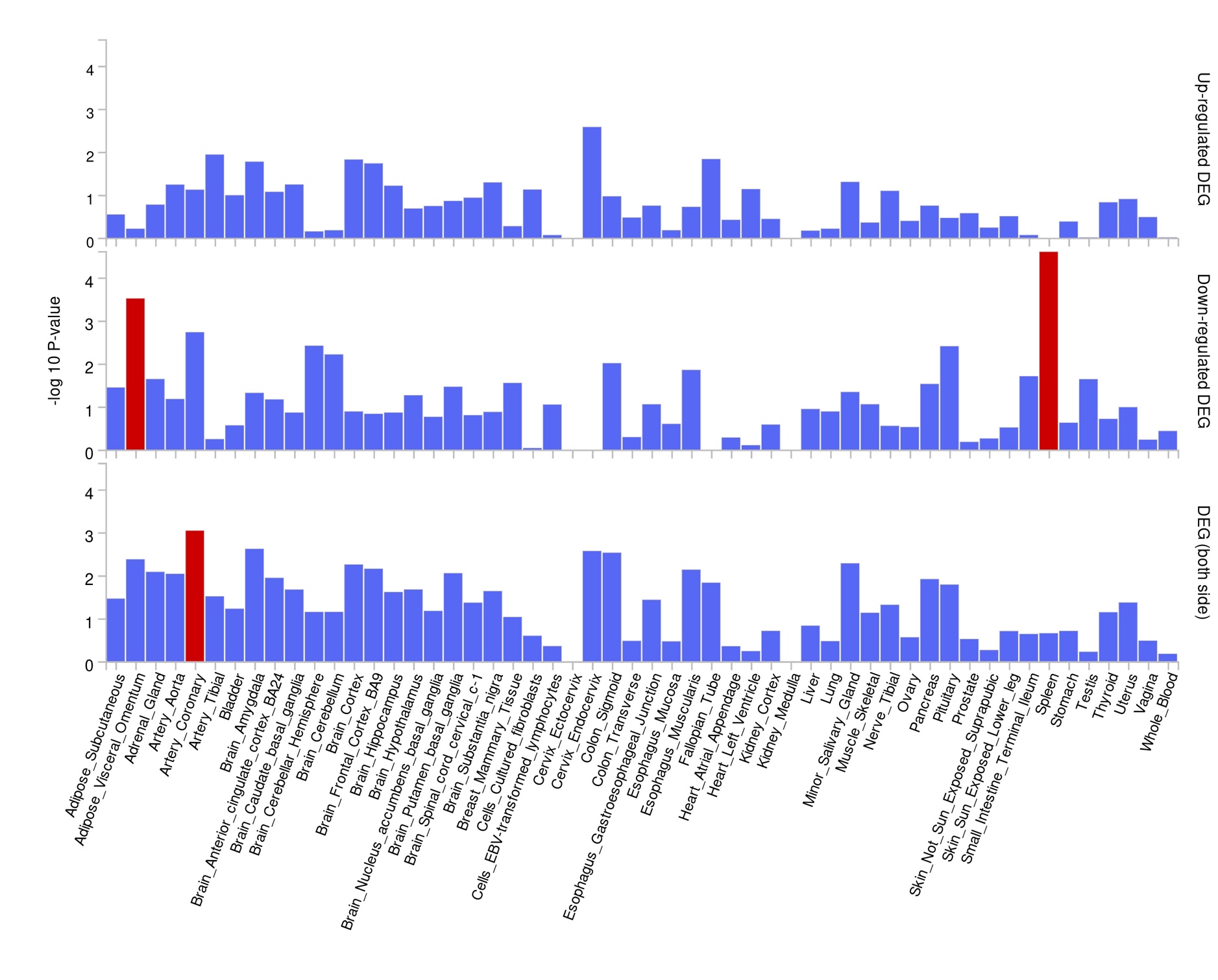


Supplementary Figure 12. Results of gene-set enrichment analysis in GTEX v8 54 tissue type dataset for genes annotated to the top 100 CpGs associated with SSRI exposure. Gene-set enrichment performed using FUMA GENE2FUNC. Bonferroni-adjusted p-values < 0.05 considered significant.


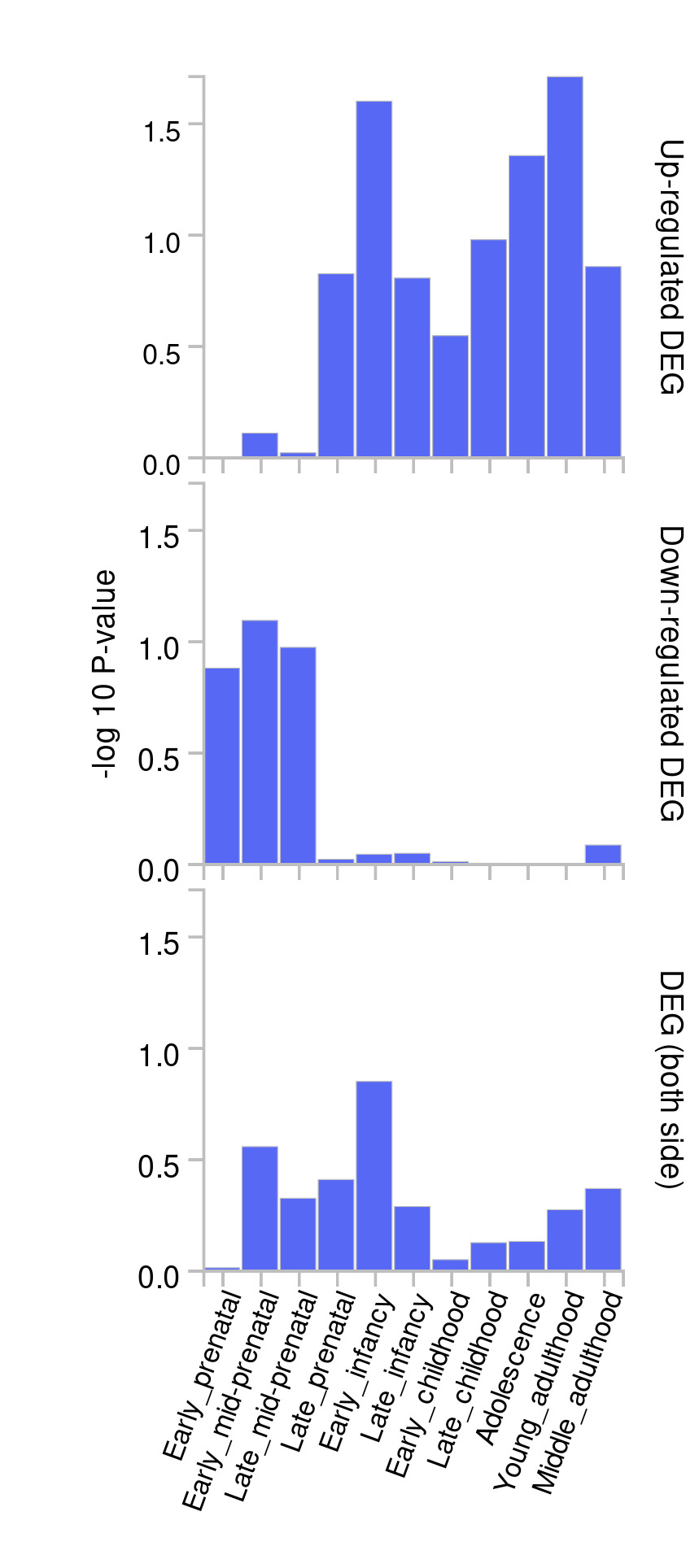


Supplementary Figure 13. Results of gene-set enrichment analysis in the BrainSpan stage of development dataset for genes annotated to the top 100 CpGs associated with SSRI exposure. Gene-set enrichment performed using FUMA GENE2FUNC. Bonferroni-adjusted p-values < 0.05 considered significant.

## Differential Expression


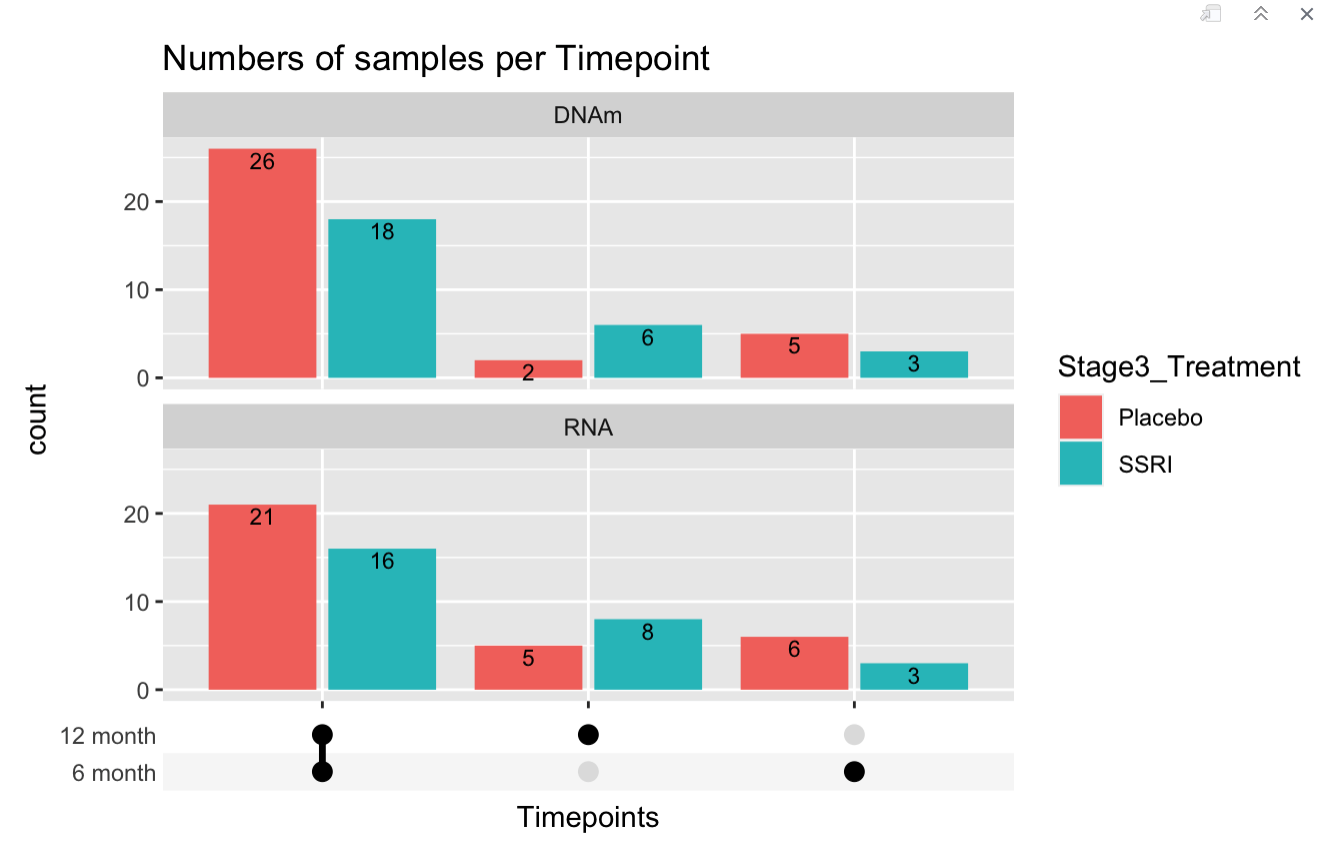


Supplementary Figure 14. Number of DNA-methylation and RNA samples provided per timepoint for each treatment group. Fewer participants provided a 6-month sample for RNA compared to DNA-methylation.


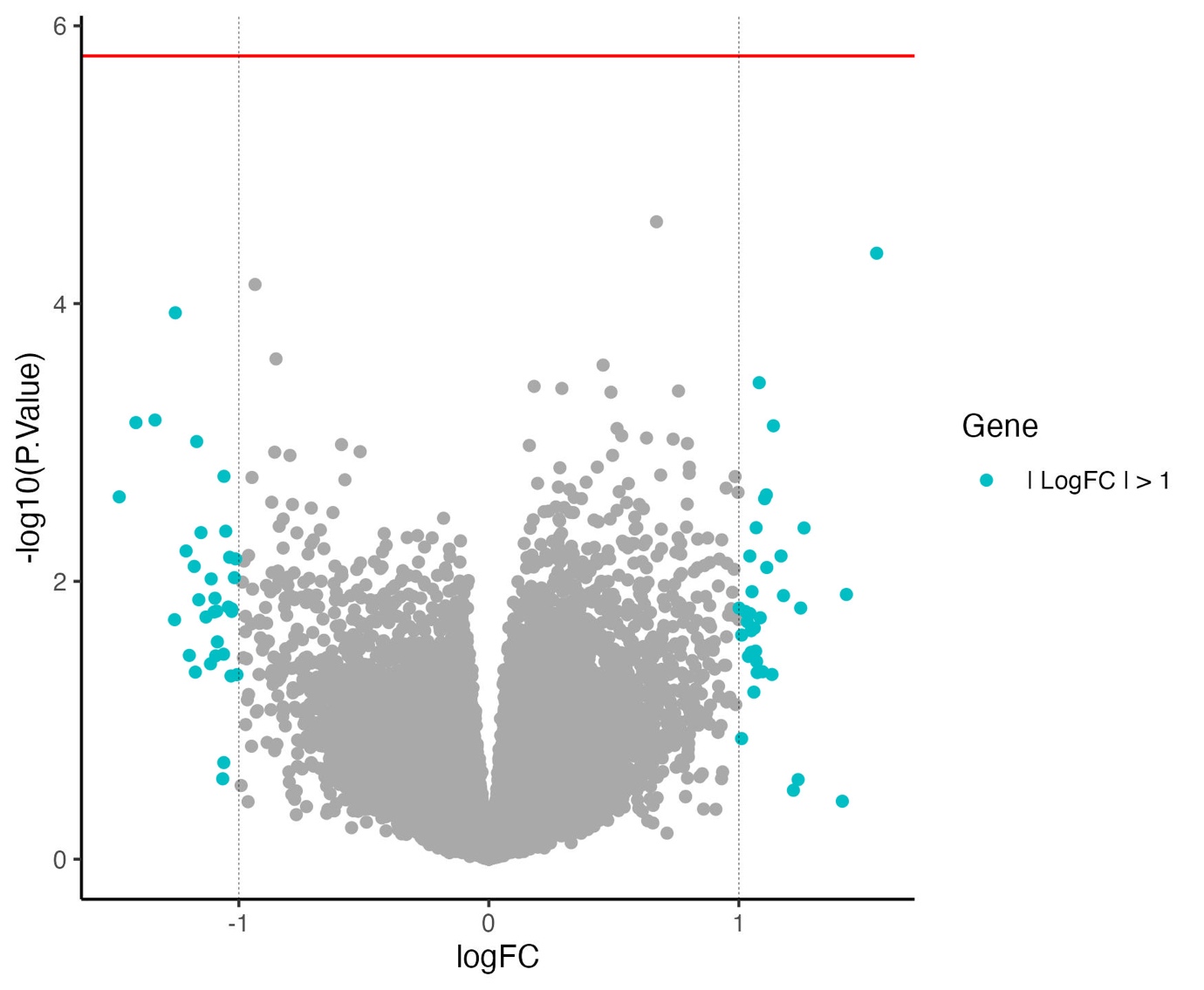


Supplementary Figure 15. Association of gene expression with the timepoint:treatment interaction term in all eligible samples. Red line represents the Bonferroni-corrected p-value significance threshold of p < 2.33 x10^-7^.


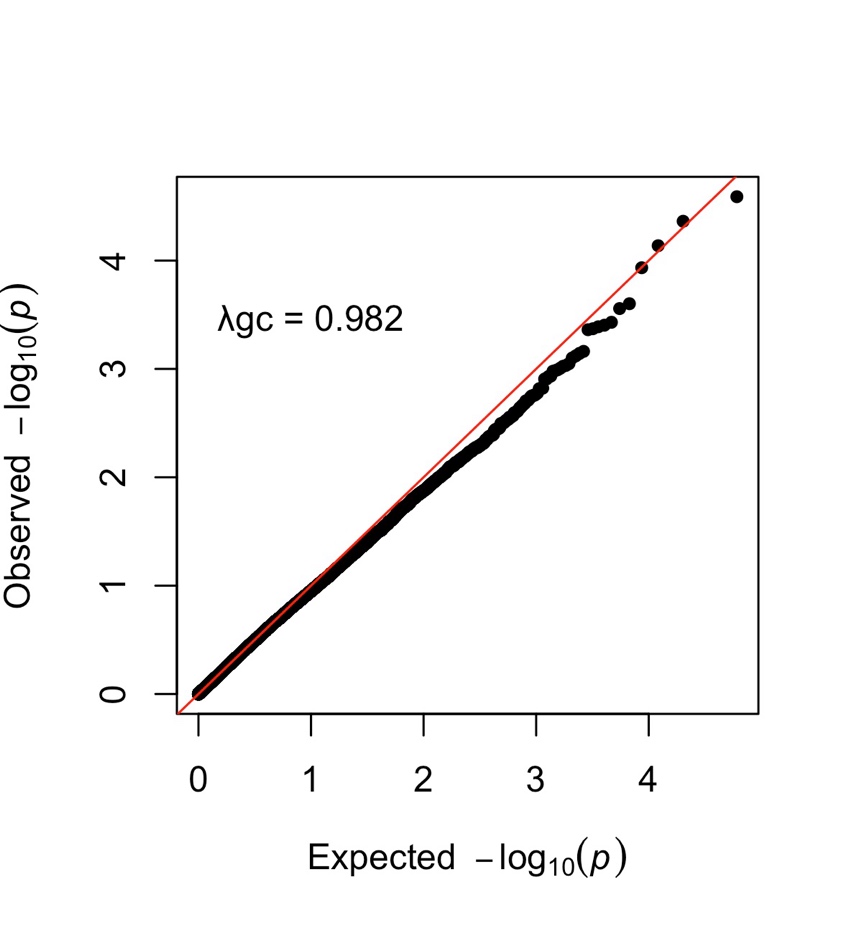


Supplementary Figure 16. QQ-plot of expected vs observed p-values of association between gene expression and the timepoint:treatment interaction term in all eligible samples. λgc is the genomic inflation factor.

## Antidepressant Methylation Profile Score

Of the 212 probes used to calculate the Davyson et al. antidepressant MPS, 209 were present in the cleaned STEP DNAm dataset. The three missing probes did not meaningfully affect the distribution of probe weights (Supplementary Figure 17).


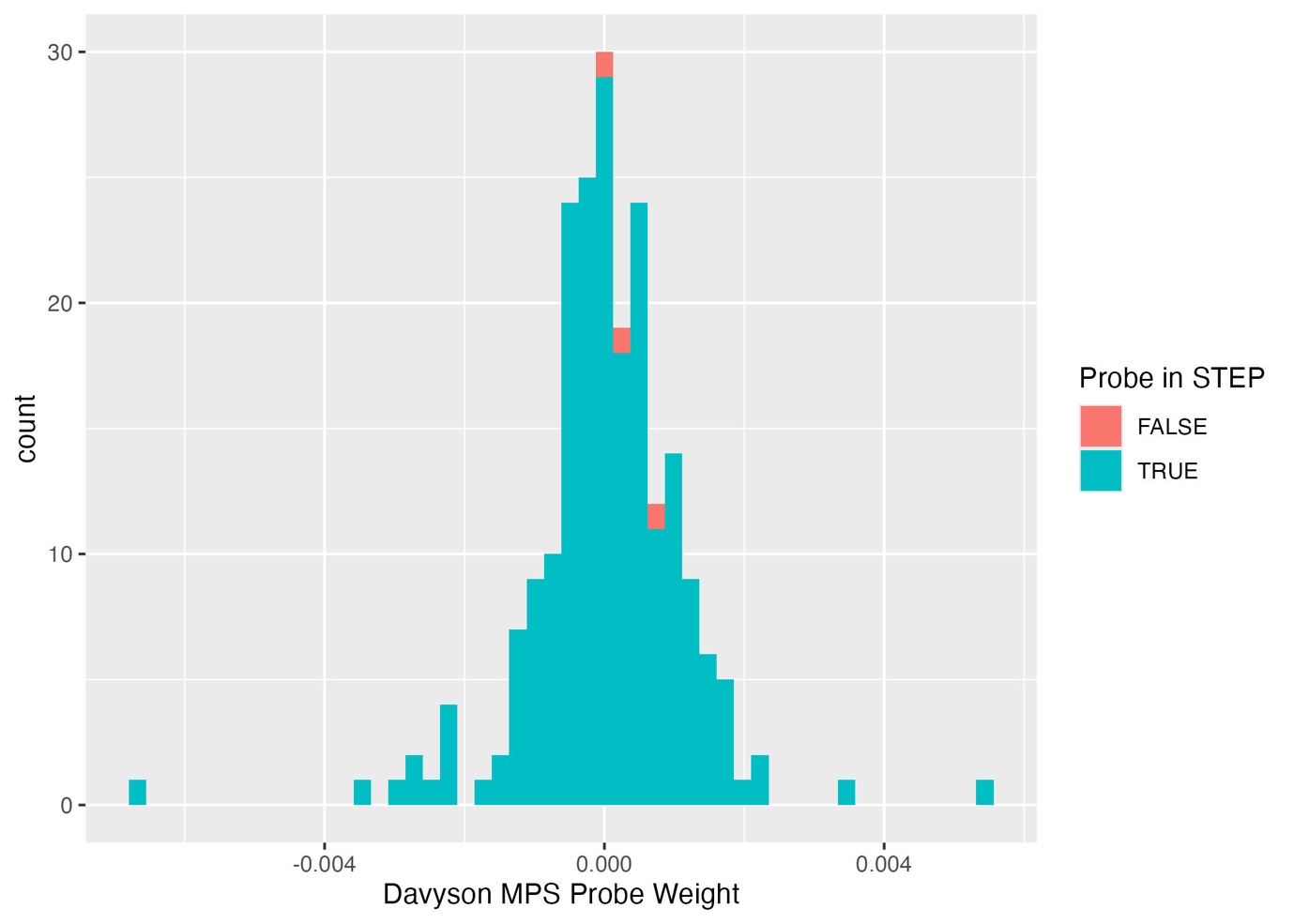


Supplementary Figure 17. Distribrution of probe weights in the Davyson et al. antidepressant MPS, coloured by whether the probe was available in the STEP DNAm dataset.


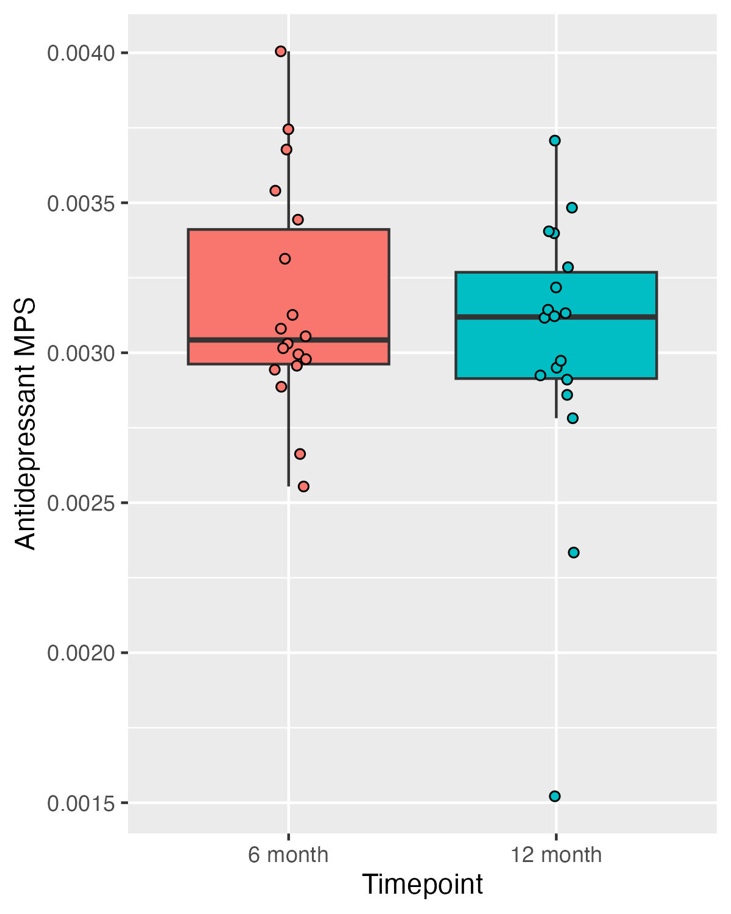


Supplementary Figure 18. Antidepressant MPS for 6-month and 12-month methylation samples from participants randomised to the SSRI treatment group. Only participants with both a 6- and 12-month sample are shown here, as these samples were used to test the performance of the MPS.

## Association of Antidepressant MPS with clinical scores

Antidepressant MPS were assessed for association with clinical scores across the 6- and 12-month timepoints using a mixed effect model with participant as a random intercept. Accordingly, only participants who provided both timepoints were included. A second model that included the timepoint*Treatment interaction was also tested, to account for potential changes in clinical measures due to treatment with SSRI. The significance threshold was defined using a Bonferroni correction for the 8 clinical measures tested (p < 0.05/8).

No clinical scores were significantly associated with the MPS in either the without-covariates model or the model including the timepoint*treatment interaction (Supplementary Figure 19).


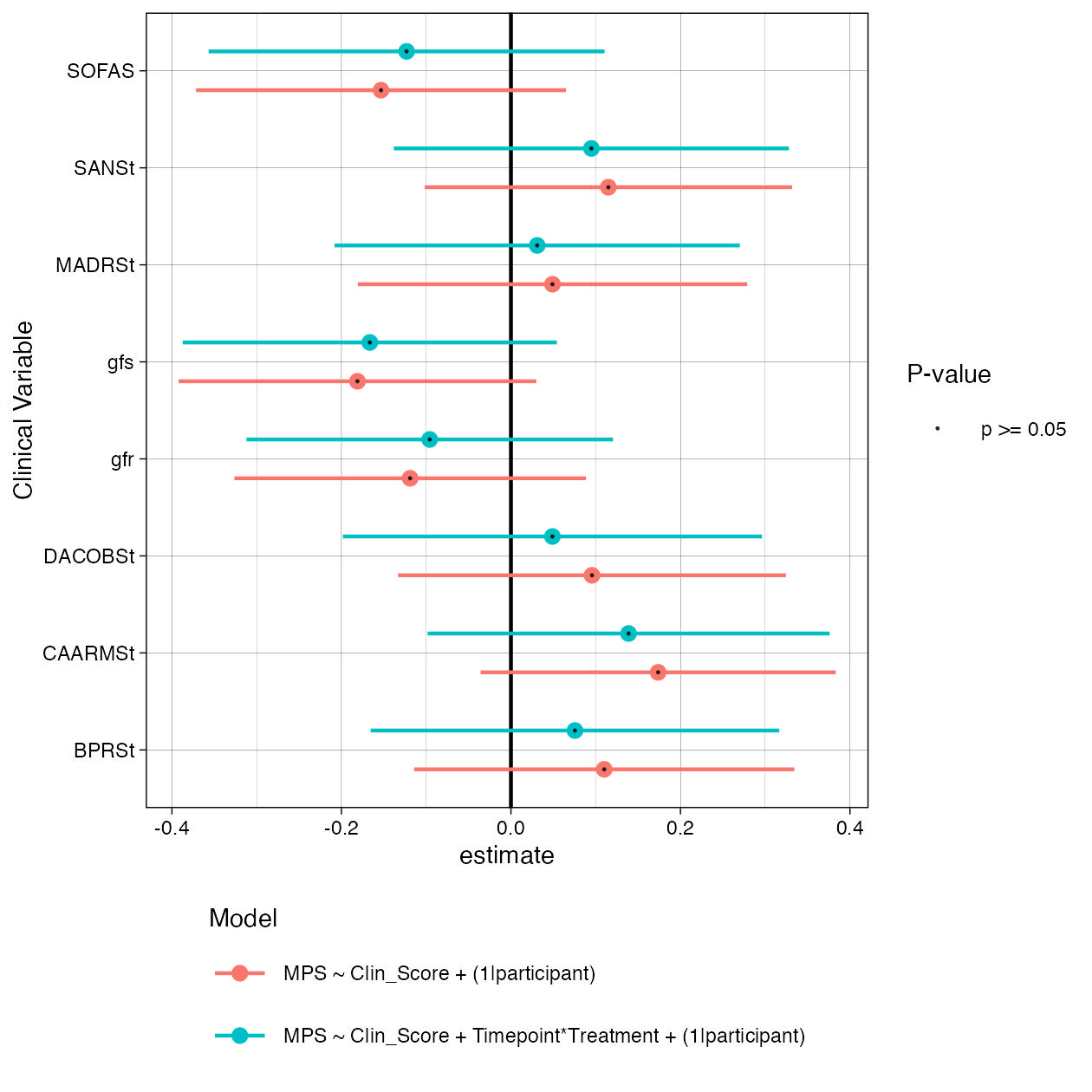


Supplementary Figure 19. Estimate and confidence intervals of association between the antidepressant MPS and scores for 8 clinical measures. Abbreviations: MADRS, Montgomery–Åsberg Depression Rating Scale; CAARMS, the Comprehensive Assessment of At-Risk Mental States; BPRS, Brief Psychiatric Rating Scale; SANS, Scale for the Assessment of Negative Symptoms; SOFAS, Social and Occupational Functioning Assessment Scale; DACOBS, Davos Assessment of Cognitive Biases Scale.

## Association of white blood cells with SSRI Exposure

We previously identified differences in DNA-methylation-derived white blood cell (WBC) proportions that anticipated remission of UHR status after treatment with psychosocial therapy^12^. Cell-type proportions derived from DNA-methylation, gene expression and clinical pathology blood tests, and cell counts from pathology blood tests, were assessed separately for association with SSRI exposure using linear regression models. SSRI exposure was represented in the regression model as the interaction of treatment group (SSRI or Placebo) and timepoint (6- or 12-months). Sex, age and two genetic PCs were included as covariates and participant was included as a random effect.

$$celltype \sim Sex + Age + Genetic PC1 + Genetic PC2 + Treatment*Timepoint + \left( 1 | participant \right)$$

Cell counts (not proportions) were log-transformed prior to analysis. Cell-types with predominantly low/zero proportions were not tested (eosinophils, basophils). A Benjamini-Hochberg-adjusted p-value less than 0.05 was considered significant.

There were no significant associations between SSRI exposure and any of the cell-type proportions or counts, regardless of method of estimation or measurement (Supplementary Figure 20).


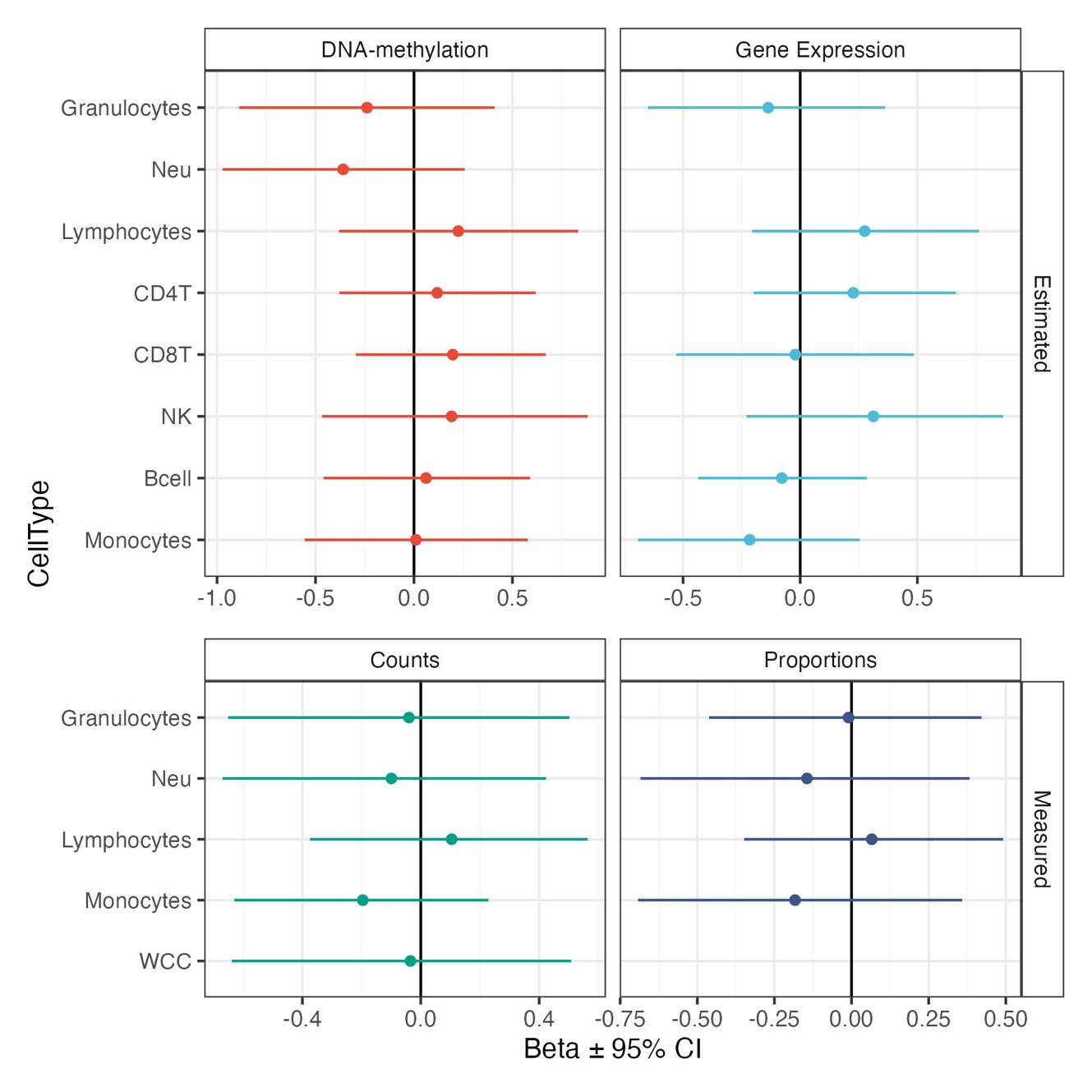


Supplementary Figure 20. Beta coefficient and 95% confidence intervals of the association of cell-type proportions with SSRI exposure. SSRI exposure is considered as the interaction between treatment group (binary, 1=SSRI, 0= Placebo) and timepoint (6-month, 12-month). Estimated proportions were estimated from DNA-methylation or gene expression samples using standard methods. Measured cell-types were measured by a registered pathology service. Neutrophils were unable to be estimated from gene expression data, and lymphocyte sub-types (CD4+ T-cells, CD8+ T-cells, Natural Killer cells, B-cells) were estimated from omics data but not part of the routine pathology screen. WCC stands for white cell count, the total count of white blood cells measured.

## Post-hoc power analysis

Post-hoc power analyses were performed using G*Power^13^, in order to ascertain the detectable effect sizes for the MWAS and differential expression analyses. The interaction between Timepoint and treatment group was approximated using a simplified design of a two-tailed t-test (i.e. modelling Placebo_change_ vs SSRI_change_). As such, the sample sizes used for each group were based on the number of participants with both timepoints. As the Benjamini-Hochberg (BH) procedure was used for multiple-testing correction, the exact significance threshold was approximated for the MWAS as the midpoint between the ranked unadjusted p-values of the last significant CpG at p_BH_<0.05 and the first non-significant CpG from the main MWAS. As no significant differentially expressed genes were identified, we calculated the Bonferroni-adjusted p-value threshold (0.05/ 30337) and then multiplied by 10, to approximate the more relaxed threshold that may have been obtained by the BH correction (no genes in our analysis had p-values low enough to be declared significant at this estimated threshold).

### MWAS G*Power results:

**t tests** - Means: Difference between two independent means (two groups)

**Analysis:** Sensitivity: Compute required effect size

**Input:** Tail(s) = Two

α err prob = 2.331386e-07

Power (1-β err prob) = 0.8

Sample size group 1 = 26

Sample size group 2 = 18

**Output:** Noncentrality parameter δ = 7.1362941

Critical t = 6.1607953

Df = 42

Effect size d = 2.1881453

### Differential Expression G*Power results:

**t tests** - Means: Difference between two independent means (two groups)

**Analysis:** Sensitivity: Compute required effect size

**Input:** Tail(s) = Two

α err prob = 1.648152e-05

Power (1-β err prob) = 0.8

Sample size group 1 = 21

Sample size group 2 = 16

**Output:** Noncentrality parameter δ = 5.9337202

Critical t = 4.9910672

Df = 35

Effect size d = 1.9690570

# Formulas used

### Univariate testing of demographic and clinical variables on Treatment group

- Wilcoxon-mann-whitney or chi-squared, no covariates

### Association of clinical measures with SSRI Exposure

$$measure \sim Sex + Age + Genetic PC1 + Genetic PC2 + Treatment*Timepoint + \left( 1 | participant \right)$$

### Association of white blood cells with SSRI Exposure

$$celltype \sim Sex + Age + Genetic PC1 + Genetic PC2 + Treatment*Timepoint + \left( 1 | participant \right)$$

### MWAS

Main analysis:

$$CpG \sim Sex + Age + Genetic PC1 + Genetic PC2 +Lymphocytes+Monocytes+Smoking MPS+ Treatment*Timepoint + \left( 1 | participant \right)$$

Sensitivity analysis on BMI (top 100 CpGs only)

$$CpG \sim Sex + Age + Genetic PC1 + Genetic PC2 +Lymphocytes+Monocytes+Smoking MPS+BMI+ Treatment*Timepoint + \left( 1 | participant \right)$$

### Differential Expression

$$gene \sim Sex + Age + Genetic PC1 + Genetic PC2 +RNAseq Pool+RNAseq array row+ Treatment*Timepoint + \left( 1 | participant \right)$$

### Antidepressant MPS

Association with SSRI exposure:

- Paired, one-sided t-test between 6- and 12-month samples from the SSRI treatment group, so no covariates.

Association with clinical scores:

$$MPS \sim Clin\_Score + (1|participant)$$

$$MPS \sim Clin\_Score + Timepoint*Treatment + (1|participant)$$

# References

1. Purcell, S. & Chang, C. PLINK. 1.9 edn.

2. Auton, A. *et al.* A global reference for human genetic variation. *Nature* **526**, 68-74 (2015).

3. Yang, J., Lee, S.H., Goddard, M.E. & Visscher, P.M. GCTA: a tool for genome-wide complex trait analysis. *Am J Hum Genet* **88**, 76-82 (2011).

4. Min, J.L., Hemani, G., Davey Smith, G., Relton, C. & Suderman, M. Meffil: efficient normalization and analysis of very large DNA methylation datasets. *Bioinformatics* **34**, 3983-3989 (2018).

5. Zhou, W., Laird, P.W. & Shen, H. Comprehensive characterization, annotation and innovative use of Infinium DNA methylation BeadChip probes. *Nucleic Acids Research* **45**, e22-e22 (2017).

6. Chen, S., Zhou, Y., Chen, Y. & Gu, J. fastp: an ultra-fast all-in-one FASTQ preprocessor. *Bioinformatics* **34**, i884-i890 (2018).

7. Dobin, A. *et al.* STAR: ultrafast universal RNA-seq aligner. *Bioinformatics* **29**, 15-21 (2013).

8. Liao, Y., Smyth, G.K. & Shi, W. The R package Rsubread is easier, faster, cheaper and better for alignment and quantification of RNA sequencing reads. *Nucleic Acids Res* **47**, e47 (2019).

9. Auwera, G.v.d. & O'Connor, B.D. *Genomics in the cloud : using Docker, GATK, and WDL in Terra*, xxiv, 467 pages (O'Reilly Media, Sebastopol, CA, 2020).

10. Mulder, R.H. *et al.* Epigenome-wide change and variation in DNA methylation in childhood: trajectories from birth to late adolescence. *Hum Mol Genet* **30**, 119-134 (2021).

11. Shen, X. *et al.* A methylome-wide association study of major depression with out-of-sample case–control classification and trans-ancestry comparison. *Nature Mental Health* (2025).

12. Barker, L.F. *et al.* White Blood Cell Proportions Are Associated With Response to Psychosocial Therapy in Young People at Ultra-High Risk for Psychosis. *Biol Psychiatry Glob Open Sci* **5**, 100546 (2025).

13. Faul, F., Erdfelder, E., Lang, A.G. & Buchner, A. G*Power 3: a flexible statistical power analysis program for the social, behavioral, and biomedical sciences. *Behav Res Methods* **39**, 175-91 (2007).
